# Coverage gaps in empiric antibiotic regimens used to treat serious bacterial infections in neonates and children in Southeast Asia and the Pacific

**DOI:** 10.1016/j.lansea.2023.100291

**Published:** 2023-10-31

**Authors:** Phoebe C.M. Williams, Mark Jones, Thomas L. Snelling, Robert Duguid, Nerida Moore, Benjamin Dickson, Yue Wu, Jessica Saunders, Priyali Wijeratne, Anousone Douangnouvong, Elizabeth A. Ashley, Paul Turner

**Affiliations:** a Faculty of Medicine, School of Public Health, The University of Sydney, Sydney, NSW, Australia; b Department of Infectious Diseases, Sydney Children's Hospital Network, Sydney, NSW, Australia; c Sydney Institute of Infectious Diseases (Sydney ID), Sydney, NSW, Australia; d Department of Infectious Diseases, Royal Darwin Hospital, Tiwi, Northern Territory, Australia; e Department of Global Health, London School of Hygiene and Tropical Medicine, London, UK; f Lao-Oxford Wellcome Trust Research Unit, Mahosot Hospital, Vientiane, Lao PDR; g Centre for Tropical Medicine and Global Health, Nuffield Department of Medicine, University of Oxford, Oxford, UK; h Cambodia Oxford Medical Research Unit, Angkor Hospital for Children, Siem Reap, Cambodia

**Keywords:** Child health, Antimicrobial resistance, WISCA, Antibiogram, Neonatal sepsis, Paediatric sepsis, Neonatal meningitis, LMIC

## Abstract

**Background:**

High levels of antimicrobial resistance (AMR) are propagating deaths due to neonatal and paediatric infections globally. This is of particular concern in Southeast Asia and the Pacific, where healthcare resources are constrained and access to newer agents to treat multidrug-resistant pathogens is limited.

**Methods:**

To assess the coverage provided by commonly prescribed empiric antibiotic regimens for children in low- and middle-income countries in Southeast Asia and the Pacific, we built a weighted incidence syndromic combination antibiogram (WISCA), parameterised using data obtained from a systematic review of published literature incorporating WHO-defined SEARO and WPRO regions in Ovid MEDLINE, EMBASE, Global Health and PubMed. Susceptibility data for bacterial pathogens were extracted to provide coverage estimates for pre-specified antibiotics (aminopenicillins, gentamicin, third-generation cephalosporins and carbapenems), reported at the regional level.

**Findings:**

6648 bacterial isolates from 11 countries across 86 papers were included in the Bayesian WISCA model, which weighted bacterial incidence and antimicrobial susceptibility of relevant isolates. Coverage provided by aminopenicillins in neonatal sepsis/meningitis was 26% (80% credible interval: 16–49) whilst gentamicin coverage was 45% (29–62). Third-generation cephalosporin coverage was only 29% (16–49) in neonatal sepsis/meningitis, 51% (38–64) in paediatric sepsis and 65% (51–77) in paediatric meningitis. Carbapenems were estimated to provide the highest coverage: 81% (65–90) in neonatal sepsis/meningitis, 83% (72–90) in paediatric sepsis and 79% (62–91) in paediatric meningitis.

**Interpretation:**

These findings reveal alarmingly high rates of resistance to commonly prescribed empirical therapies for neonatal and paediatric sepsis and meningitis in the Asia–Pacific region.

**Funding:**

This research was funded in whole, or in part, by the 10.13039/100010269Wellcome Trust [220211]. For the purpose of Open Access, the author has applied a CC BY public copyright licence to any Author Accepted Manuscript version arising from this submission. PCMW is supported by a 10.13039/501100000925National Health and Medical Research Council (NHMRC) Investigator Grant. NHMRC had no involvement in the design or conduct of the research.

**Research in context**

**Evidence before this study**

There is increasing evidence of high levels of resistance to commonly prescribed antibiotics to treat sepsis and meningitis in neonates and children globally. This is of particular concern in resource-constrained settings, where access to more efficacious regimens to treat multidrug-resistant infections is limited.

**Added value of this study**

To assist clinicians and policymakers to understand the burden of AMR in children in the populous Southeast Asia and Pacific regions, we performed a systematic search of the literature to build a weighted incidence combination antibiogram (WISCA), parameterised using susceptibility data from 6648 bacterial isolates collated from 11 countries. Coverage provided by commonly prescribed antibiotics to treat sepsis and meningitis in children and neonates – aminopenicillins, gentamicin and third-generation cephalosporins – was low. Carbapenems provided higher rates of coverage, yet their widespread use needs to be balanced against propagating further AMR, particularly carbapenem-resistant infections.

**Implications of all the available evidence**

Currently recommended empirical regimens for neonates and children with sepsis and meningitis are providing limited coverage in Southeast Asia and the Pacific. New regimens with improved efficacy to treat these common infectious conditions in children are urgently needed. Children and neonates should be prioritised in future interventional trials to evaluate novel empirical therapy regimens with improved efficacy to treat meningitis and sepsis globally.

## Introduction

Antimicrobial resistance (AMR) is one of the greatest threats to human health of the 21st century.^1^ Given the high burden of infectious diseases affecting children and neonates, the paediatric population is most significantly affected by growing rates of non-susceptibility to commonly prescribed antibiotics. In neonates alone, an estimated 3 million cases of sepsis occur each year, resulting in up to 570,000 sepsis-attributable deaths – many of which are due to resistance to currently recommended and available antibiotics.^2^

Most deaths in young children remain in low- and middle-income (LMIC) countries, and a number of recent systematic reviews reveal a high level of bacterial resistance to World Health Organization (WHO)-recommended empirical treatments for bloodstream infections in children in LMICs.^3^, ^4^, ^5^ Asia has been identified as a particularly vulnerable geographic region for avoidable neonatal and child mortality.^6^ Despite this, there is a paucity of data published to understand the epidemiology and non-susceptibility rates for serious bacterial infections in the region; which is of particular concern given the predominance of gram-negative bacteria causing invasive infections in Asian countries.^7^

Bloodstream infections (BSIs) and meningitis are associated with significant morbidity and mortality in neonates and children, and require prompt and efficacious empiric antibiotic therapy to prevent adverse outcomes – ideally followed by targeted therapy once the results of culture and susceptibility testing are available.^8^ However, this is rarely possible in many healthcare settings, particularly in LMICs. Culture-negative bacterial infections are common in children, due to procedural challenges of blood culture collection, and frequent pre-culture exposure to antibiotics that might be prescribed intrapartum or in the community. Limited availability of blood culture analyses in many healthcare settings further compounds these diagnostic challenges, limiting the ability to prescribe directed antibiotic therapy.^9^

Consequently, most serious bacterial infections in neonates and children rely on empirical antibiotic therapy, guided by global policies that often fail to consider the local prevalence of causative pathogens, or increasing AMR.^10^ For paediatric and neonatal BSIs and meningitis, the WHO currently recommends ampicillin and gentamicin, or third-generation cephalosporins (cefotaxime, ceftriaxone) as first-line therapy.^11^ However, there is mounting evidence to suggest that the most common bacteria responsible for sepsis and meningitis in children are frequently resistant to these empirical regimens, resulting in excess mortality due to treatment with inefficacious therapies.^12^^,^^13^

Knowledge of the coverage provided by alternative antibiotic regimens for specified clinical syndromes can assist clinicians (and policy-making bodies) to select empirical antibiotic regimens with optimal efficacy.^14^ While single institution antibiograms help provide local ‘drug-bug’ treatment guidance, the data required for antibiograms are scarce in resource-constrained settings and require regular revision due to rapidly evolving rates of AMR. Antibiograms are also unable to provide syndrome-specific estimates of coverage, given their failure to consider the causative pathogens responsible for clinical syndromes – which vary substantially by age group.^15^

To improve the utility of antibiograms and address the limited availability of published susceptibility data, the Weighted Incidence Syndromic Combination Antibiogram (WISCA) methodology has been developed.^16^ WISCAs aim to inform the antibacterial coverage of specified antibiotic regimens for a given clinical syndrome, using estimates of both the prevalence of bacterial pathogens causing that syndrome and the antibiotic susceptibility of these pathogens.^15^^,^^16^ Bayesian methods are used to calculate a weighted coverage estimate for individual or pre-specified combinations of antibiotics, following pre-identified susceptibility assumptions consistent across the international literature.^16^, ^17^, ^18^, ^19^

WISCA coverage estimates can inform the proportion of episodes of a given clinical syndrome that would be expected to be treated with specified antibiotic regimens, even without knowledge of the causative pathogen or its specific susceptibility, by incorporating the frequencies of different bacterial pathogens (and their intrinsic resistance patterns) into the model.^16^^,^^18^^,^^19^ WISCAs may be particularly helpful for informing the empirical treatment of sepsis and meningitis – where pathogen isolation may be difficult and the consequences of ineffective therapy are high – whilst also providing a tool for antibiotic stewardship, which is of importance in light of increasing global AMR.^1^^,^^5^^,^^15^^,^^20^

As a demonstration of this approach, we have developed a WISCA for sepsis and meningitis in the paediatric population – the most important causes of neonatal and child mortality globally - in a region with limited surveillance capacity and limited published data.^7^^,^^21^ Our objective was to use published data to evaluate the coverage offered by commonly recommended and prescribed antibiotics to treat sepsis and meningitis in children in Southeast Asia and the Pacific: ampicillin, gentamicin, non-antipseudomonal third-generation cephalosporins (ceftriaxone, cefotaxime) and carbapenems. Utilising this strategy, we aim to provide insight and understanding into the likelihood that empirical antibiotic regimens are able to provide effective coverage against serious bacterial infections children in the region. These findings can inform the need for development of new empirical regimens with improved coverage against the pathogens most commonly responsible for sepsis and meningitis in children in the context of increasing AMR.^10^

## Methods

### Data collection

A systematic search of the literature was conducted between January and August 2021 in PubMed, OVID MEDLINE, Global Health and EMBASE databases for each region, using both free-text and MeSH terms (PROSPERO: CRD42021248722 and CRD4202125930). Titles and abstracts were screened for inclusion by two independent reviewers and where disagreement existed, these were resolved by an independent third reviewer. All full-text articles were assessed for inclusion and deemed relevant if they met the pre-defined inclusion and exclusion criteria (Supplementary Table 1) as demonstrated in the PRISMA flow diagram (Supplementary Fig. 1). A citation search was conducted to identify additional studies (grey literature) by reviewing reference lists of publications eligible for full-text review. The Grades of Recommendation, Assessment, Development and Evaluation Working Group (GRADE)^22^ and Microbiology Investigation Criteria for Reporting Objectively (MICRO) framework^23^ were applied to summarise the quality of evidence for each study by two authors; any discrepancies were resolved via consensus or engagement of a third reviewer.

The search was restricted to countries in the WHO-defined WPRO and SEARO regions, and excluded those classified as World Bank-defined high-income countries.^24^ To ensure estimates were contemporaneous, we limited the search to articles published between 2011 and 2021. Studies were reviewed against pre-specified eligibility criteria and data were extracted into a standardised spreadsheet (Supplementary Table 2). Extracted data included: details of the study site and patient population, the study design, calculation of the total number of bacterial isolates from relevant blood or cerebrospinal fluid (CSF) cultures, the number of isolates of specific bacterial species known to cause the relevant infectious syndrome, the number of isolates tested for susceptibility to the antibiotics relevant for establishing coverage offered by the prespecified regimens of interest, and the number of isolates found to be susceptible to these antibiotics. In the absence of detailed clinical information, we excluded bacteria more frequently associated with contamination rather than true infection (specifically, coagulase-negative staphylococci), in keeping with methodology used in other similar studies.^17^

### Selecting clinically relevant bacteria and syndromes

Pre-specified bacterial pathogens were selected that included the Global Antimicrobial Resistance and Use of Surveillance System (GLASS) bacteria, plus other pathogens relevant to neonatal and paediatric BSI and meningitis (Table 1).^25^ These pathogens were chosen based on the subject matter expertise of the clinicians in the study team as they are likely to be most relevant to significant infections in children, and are problematic in terms of emerging AMR. Neonatal sepsis and meningitis were considered collectively given that these clinical conditions frequently co-occur, while paediatric sepsis and meningitis were treated as distinct clinical syndromes.

**Table 1:** Pre-defined pathogens for inclusion.

Bacterial GLASS 2020 organisms 1. *Escherichia coli* 2. *Klebsiella pneumoniae* 3. *Acinetobacter* spp. 4. *Staphylococcus aureus* 5. *Streptococcus pneumoniae* 6. *Salmonella* spp. 7. *Shigella* spp. 8. *Neisseria gonorrhoeae* 9. *Pseudomonas aeruginosa* P**lus** other bacterial pathogens of particular importance for invasive infections in children: 10. *Streptococcus agalactiae* 11. *Streptococcus pyogenes* 12. *Haemophilus influenzae* 13. *Neisseria meningitidis*

### Regimens selected for coverage estimation

The three regimens evaluated in this study were aminopenicillins, gentamicin (WHO recommended first-line treatment for neonatal sepsis), non-antipseudomonal third generation cephalosporins (ceftriaxone and cefotaxime: WHO recommended first-line therapy for neonatal meningitis and paediatric sepsis, or second-line therapy for neonatal sepsis), and carbapenems (as meropenem, classified by the WHO as a ‘Watch’ antibiotic, is frequently prescribed as empiric therapy in many clinical settings due to increasing non-susceptibility to first- and second-line therapies).^10^^,^^26^

### Intrinsic resistance and combination regimens

Pathogens known to have intrinsic *in vivo* resistance to certain antimicrobials were allocated a susceptibility rate of zero, regardless of any contrary published data. These included aminopenicillins for *Klebsiella* spp., cephalosporins for *Enterococcus* spp., cefotaxime/ceftriaxone for *Pseudomonas* spp., aminopenicillins and ceftriaxone/cefotaxime for *Acinetobacter* spp., and gentamicin for *Salmonella* spp. and *Streptococcus pneumoniae* (Supplementary Tables 2 and 3).^27^, ^28^, ^29^

### Parameter estimation & WISCA model

The statistical approach for WISCA comprises independent Bayesian models for estimating the distribution of causative pathogens and the susceptibility of the bacteria to the antibiotics considered in this study. The former used a Multinomial-Dirichlet conjugate model, and the latter used a binomial logistic regression.^30^ Both models utilised regularising priors, and the latter incorporates variance components. The specifications for each model along with a more detailed description can be found in the supplementary materials (Supplementary Table 4 - model specifications). Based on the joint posterior distributions, we computed the coverage estimates for each syndrome separately by extracting Markov chain Monte Carlo (MCMC) samples from the relevant prevalence and susceptibility models and producing a prevalence weighted view of susceptibility, which were then totalled to provide the coverage by antibiotic classification.

In calculating susceptibility, assumptions as outlined above were incorporated, including those pertaining to the intrinsic resistance of organisms and susceptibility across antibiotic classes, when susceptibility testing was lacking (specified in Supplementary Table 2).^20^ The models were fit using Hamiltonian Monte Carlo using 3 chains and 20,000 draws per chain for all models. Convergence was checked via traceplots and Gelman-Rubin (R-hat) statistics. All models were implemented in Stan and executed via the cmdstanr package within the R programming environment (R Core Team, 2023).^31^, ^32^, ^33^

### Role of the funding source

PCMW is supported by an NHMRC Investigator Grant (119735). NHMRC had no involvement in the design or conduct of the research. This research was funded in whole, or in part, by the Wellcome Trust [220211]. Wellcome Trust had no involvement in the design or conduct of the research.

## Results

### Description of the data set

The systematic review yielded data from 103 publications from the WHO-defined Southeast Asia Region (SEARO) and 48 publications in the WHO-defined Western Pacific Region (WPRO), representing 11 countries in total. Of these, 65 papers were focussed on only a single pathogen, and were therefore excluded to minimise bias in estimating the prevalence of pathogens responsible for relevant syndromes. Subsequently, data from 86 papers were extracted for incorporation into the WISCA tool (Supplementary Fig. 1). This included clinical data from a variety of clinical contexts across non-governmental and tertiary urban units; only one paper^34^ analysed a rural population. The data from these publications were collated across a period that encompassed 1990–2019.

Nine papers (of 86, 10%) specified that their data pertained to hospital-acquired infections alone, while 46 (53%) papers analysed neonatal sepsis alone and the remaining publications incorporated data from both neonatal and paediatric age groups. Only one paper was GRADE level A (high quality) evidence,^35^ four papers^36^, ^37^, ^38^, ^39^ were ranked as GRADE level B (moderate-quality) evidence, and the remaining were ranked as low or very low-quality evidence. Eight papers received the lowest MICRO grade (E) whilst the remainder were MICRO grade C or D; only two papers^35^^,^^37^ ranked MICRO level B (Supplementary Table 2).^23^

### Causative pathogens

**Fig. 1:**
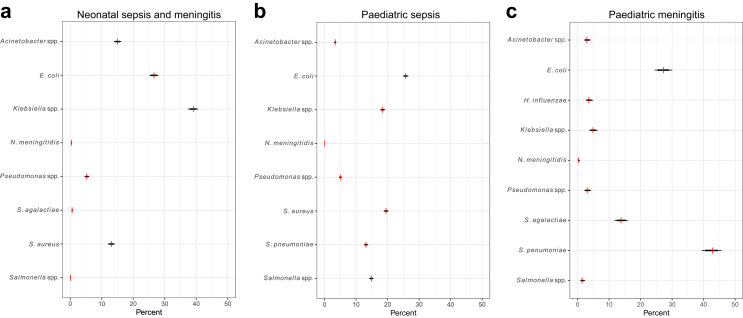
Distribution of causative pathogens within each clinical syndrome. a. Neonatal sepsis/meningitis; b. Paediatric sepsis (children >1 month of age); c. Paediatric meningitis (children >1 month of age). The red vertical lines show the observed proportions of each pathogen responsible for each clinical syndrome, as summarised from data in the literature, whilst the black horizontal lines indicate the 80% (thick lines) and 99% (thin lines) credible intervals.

The distribution of pathogens reported within each pre-defined syndrome is summarised in Fig. 1.

The most common pathogens isolated in neonatal sepsis/meningitis were *Klebsiella* spp. (39%, 1337 isolates) and *Escherichia coli* (27%, 910 isolates), with *Streptococcus agalactiae* only reported in 1% of cases (20 isolates). *Acinetobacter* spp. and *Staphylococcus aureus* were also isolated in a sizeable proportion of neonatal sepsis cases (515 [15%] and 447 [13%] of cases, respectively).

In paediatric sepsis, *E. coli* (26%, 2538 isolates), *S. aureus* (20%, 1924 isolates) and *Streptococcus pneumoniae* (13%, 1298 isolates) were the most commonly isolated pathogens; *Salmonella* spp. (15%, 1464) and *Klebsiella* spp. (18%, 1816 isolates) were also reported. This is similar to the profile of pathogens identified in cases of paediatric meningitis, with *S. agalactiae* (14%, n = 136) also isolated in a sizeable proportion of cases (predominantly occurring in the post-neonatal period) whilst *S. pneumoniae* was the most important cause of paediatric meningitis in older children (43%, 421 isolates) followed by *E. coli* (27%, 269 isolates).

### Parameter values: isolates reported and susceptibility

In total, antimicrobial susceptibility data for 3423 isolates were evaluated in neonatal sepsis/meningitis: 9866 in paediatric sepsis and 984 in paediatric meningitis. Some pathogens were over-represented by certain countries – for example, 32% of all *E. coli* isolates arose from research conducted in China, whilst 33% of all *Klebsiella* spp. isolates arose from research conducted in India. Table 2 reveals the susceptibility estimates revealed using the modelling approach detailed above, excluding pre-specified intrinsically-resistant combinations. Susceptibility was not necessarily performed against the same antibiotics in all studies, but was frequently inferred (for example, utilising oxacillin/cefoxitin results to infer susceptibility to β-lactams in *S. aureus*) in keeping with standardised microbiological processes.^40^

**Table 2:** Susceptibility estimates by pathogen.

Pathogen	Antibiotic	Susceptibility estimates (Posterior median; 80% credible interval)
*Acinetobacter* spp.	Carbapenems	0.54 (0.10, 0.93)
*Escherichia coli*	Aminopenicillins	0.16 (0.04, 0.47)
*Escherichia coli*	Gentamicin	0.47 (0.17, 0.80)
*Escherichia coli*	Third-generation cephalosporins	0.36 (0.11, 0.72)
*Escherichia coli*	Carbapenems	0.94 (0.78, 0.99)
*Haemophilus influenzae*	Aminopenicillins	0.46 (008, 0.86)
*Haemophilus influenzae*	Gentamicin	0.95 (0.68, 0.99)
*Haemophilus influenzae*	Third-generation cephalosporins	0.95 (0.66, 0.99)
*Haemophilus influenzae*	Carbapenems	1.00 (0.95, 1.00)
*Klebsiella* spp.	Gentamicin	0.49 (0.12, 0.87)
*Klebsiella* spp.	Third-generation cephalosporins	0.23 (0.03, 0.73)
*Klebsiella* spp.	Carbapenems	0.89 (0.51, 0.99)
*Neisseria meningitidis*	Aminopenicillins	0.34 (0.07, 0.75)
*Neisseria meningitidis*	Third-generation cephalosporins	0.98 (0.90, 1.00)
*Neisseria meningitidis*	Carbapenems	0.94 (0.59, 1.00)
*Pseudomonas* spp.	Gentamicin	0.78 (0.41, 0.95)
*Pseudomonas* spp.	Carbapenems	0.79 (0.43, 0.95)
*Salmonella* spp.	Aminopenicillins	0.61 (0.08, 0.96)
*Salmonella* spp.	Third-generation cephalosporins	0.97 (0.68, 1.00)
*Salmonella* spp.	Carbapenems	0.99 (0.84, 1.00)
*Streptococcus agalactiae*	Aminopenicillins	0.99 (0.98, 1.00)
*Streptococcus agalactiae*	Third-generation cephalosporins	0.98 (0.95, 0.99)
*Streptococcus agalactiae*	Carbapenems	0.99 (0.93, 1.00)
*Streptococcus pneumoniae*	Aminopenicillins	0.92 (0.66, 0.99)
*Streptococcus pneumoniae*	Third-generation cephalosporins	0.87 (0.54, 0.98)
*Streptococcus pneumoniae*	Carbapenems	0.67 (0.26, 0.92)
*Staphylococcus aureus*	Aminopenicillins	0.34 (0.08, 0.77)
*Staphylococcus aureus*	Gentamicin	0.78 (0.39, 0.95)
*Staphylococcus aureus*	Third-generation cephalosporins	0.62 (0.20, 0.91)
*Staphylococcus aureus*	Carbapenems	0.87 (0.51, 0.98)

Note: Third-generation cephalosporins includes ceftriaxone and cefotaxime.

**Fig. 2:**
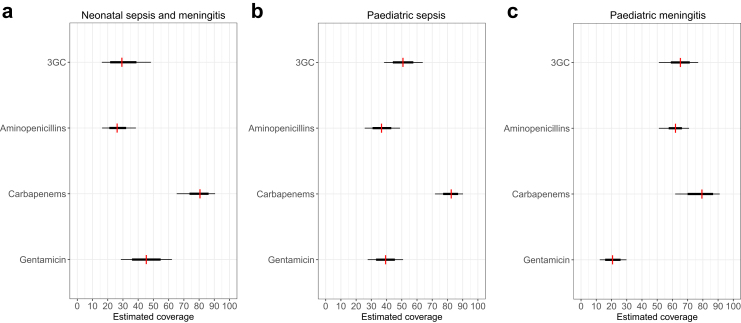
Coverage estimates by syndrome and antibiotic. Note: 3GC = Third-generation cephalosporins (ceftriaxone, cefotaxime); aminopenicillin incorporates ampicillin and amoxicillin non-susceptibility data, and carbapenem incorporates published non-susceptibility data for both meropenem and imipenem. The thin and thick lines correspond to the 80% and 50% credible intervals.

Fig. 2 and Table 3 provide the syndrome-specific estimates for coverage, which is established by weighting the susceptibility estimates by the proportional attribution of pathogens to the syndrome. In neonatal sepsis and meningitis, we estimated the coverage provided by aminopenicillins was 26% (80% CrI: 16–39), gentamicin was 45% (29–62), cefotaxime/ceftriaxone was 29 (16–49), and carbapenems 81 (65–90). For paediatric sepsis, coverage provided by aminopenicillins was 37% (26–49), gentamicin 39% (28–51), cefotaxime/ceftriaxone 51% (38–64), and carbapenems 83 (72–90). For paediatric meningitis, coverage estimates provided by aminopenicillins was 62% (51–71), gentamicin 21% (12–30), cefotaxime/ceftriaxone 65% (51–71), and carbapenems 79% (62–91).

**Table 3:** Coverage estimates (posterior median and 80% credible interval) by antibiotic and clinical syndrome.

Antibiotic	Neonatal sepsis/meningitis	Paediatric sepsis (>1 month)	Paediatric meningitis (>1 month)
Aminopenicillin	26 (16, 39)	37 (26, 49)	62 (51, 77)
Gentamicin	45 (29, 62)	39 (28, 51)	21 (12, 30)
Third-generation cephalosporins	29 (16, 49)	51 (38, 64)	65 (51, 77)
Carbapenems	81 (65, 90)	83 (72, 90)	79 (62, 91)

Note: Third-generation cephalosporins includes ceftriaxone and cefotaxime.

## Discussion

Based on a systematic review of contemporaneously published studies and utilising a Bayesian modelling approach, we estimated the coverage provided by frequently recommended empirical antibiotic regimens for the treatment of neonatal sepsis/meningitis, paediatric sepsis, and paediatric meningitis. Our analysis suggests that there are very high rates of non-susceptibility to currently recommended empirical antibiotic regimens used to treat serious bacterial infections in children in LMICs in the Asia–Pacific region. This is of particular concern given this is a region of the world where these clinical syndromes cause significant morbidity and mortality, and where AMR is increasing.^5^

Data from 86 papers incorporating 6648 isolates were pooled to predict the treatment coverage of common empirical regimens, which provides the opportunity to maximise the information provided by the available (yet unacceptably sparse) published data; whilst also informing clinicians and policy makers of the growing burden of AMR evident in the region, alongside the pressing need for improved access to effective therapy to treat multidrug-resistant (MDR) infections in children. The uncertainty of the estimates is wide and likely under-estimated, indicating the need for more robust, systematically-collated and publicly-available data in this field.

This study is based on a wide range of publications from which we have used the data to infer susceptibility profiles and the distribution of pathogens. The models used may not have addressed all sources of variability in the data, and include a level of outcome reporting bias. Nevertheless, the results represent an important approximation of coverage for a setting where robust data sources are severely limited.

For neonatal sepsis/meningitis, which was most frequently caused by gram-negative bacteria (in particular, *Klebsiella* spp. and *E. coli* – concurring with many recent international observational studies),^41^, ^42^, ^43^, ^44^ optimal coverage was provided by carbapenems. However, in light of increasing carbapenem-resistant gram-negative infections globally (particularly *Acinetobacter baumannii* outbreaks in hospitalised settings),^41^ our results should promote both judicious use of carbapenems (as a ‘Watch’ antibiotic class), whilst also ensuring that critically-ill children receive access to efficacious therapy promptly in the setting of such high rates of resistance to current empirical therapies.^45^^,^^46^

For paediatric sepsis, which is frequently caused by *E. coli*, *S. aureus*, *S. pneumoniae* and *Salmonella* spp. in the Asia–Pacific, coverage provided by third-generation cephalosporins was low (51%, 38–64). Once again, improved coverage was provided by carbapenems (83%, 72–90), which also provided the highest level of coverage for paediatric meningitis (most frequently caused by *S. pneumoniae* and *E. coli*). Our data also revealed a significant burden of late-onset Group B streptococcus (*S. agalactiae*) disease, which was responsible for 14% (136/984) of paediatric meningitis cases (>1 month of age).

The WISCA approach enables coverage estimates to inform local, national and international guidelines for the treatment of serious bacterial infections in neonates and children. Such information is of particular value in the context of constrained laboratory capacity in many healthcare settings, and for clinical syndromes where timely identification of causative pathogens for individual cases can be challenging. The high rates of non-susceptibility to empirical antibiotic options suggested by our coverage estimates indicate that a move away from current recommendations may be needed, and that guidance might instead need to be stratified by region and clinical syndrome; whilst also taking into account patient-level factors: such as the location (hospital or community) of infection acquisition, the presence of comorbidities or known colonisation with MDR organisms.

Whilst these coverage estimates are informative for understanding rates of non-susceptibility to currently recommended first- and second-line antibiotic regimens, it is important to note that widespread use of carbapenem-containing antibiotic regimens may propagate gram-negative resistance mechanisms. This is increasingly important in the context of a sparse global antibiotic development pipeline – particularly for MDR gram-negative infections in children, for whom available treatment options, particularly in LMIC settings, are extremely limited.^47^

WISCA methods extend the framework of classic bug-drug antibiograms; however there remain important methodologic challenges in applying this approach, especially where there is a paucity of high-quality published data available. The data collated by our systematic review largely arose from urban tertiary hospital settings with over-representation from particular countries (especially India and China), and is therefore not necessarily representative of community-acquired infections in rural settings in Southeast Asia and the Pacific. Furthermore, the limited published data available was of low microbiological quality (as assessed against the MICRO framework),^23^ indicating insufficient consistency across microbiological methodology, whilst also contributing to sampling and reporting biases.

Other considerations in interpreting conventional antibiograms and WISCA estimates include the challenges of extrapolating *in vitro* data to draw *in vivo* assumptions.^16^ By improving the standardised reporting of routine microbiological and clinical surveillance data and ensuring open access to underlying data sources, these inherent challenges to antibiogram and WISCA methodology (and their subsequent clinical and policy-guiding utility) could be substantially improved.^48^

Our analysis has revealed that the geographically imbalanced and sparse, low-quality data available to inform the development of coverage estimates of routine empirical antibiotics across Southeast Asia and the Pacific indicates the urgent need for more robust, systematically collated prospective surveillance data to better understand the causes of serious bacterial infections in children and neonates in LMIC settings.^48^ In light of the growing burden of AMR in these settings, an urgent focus on improving our understanding of the available efficacious therapies that may reduce the morbidity and mortality of serious infections in children is required. Whilst we wish to emphasise that our approach is exploratory and amounts to a first approximation, these data call into urgent question the adequacy of coverage currently provided by WHO-recommended first- and second-line antibiotic regimens. Evaluation of potential alternative antibiotic regimens, targeted to local pathogen and AMR patterns, will be essential to reduce unnecessary morbidity and mortality for children in this region; whilst ensuring antimicrobial stewardship is optimised in the context of rapidly growing AMR worldwide, and ensuring antibiotic development programs promptly promote access to new agents to treat MDR infections in children globally.^49^

## Contributors

PCMW, EAA and PT conceptualised the research, MJ, TLS and YW built the methodology behind the WISCA tool and evaluated the susceptibility data to provide coverage estimates. PT and EAA provided significant microbiological input in building the WISCA model, including prior assumptions. PCMW, JS, RD, BD, PW and AD performed the literature search and reviewed papers for inclusion alongside performing data extraction of included papers. PW, RD and NM conducted GRADE and MICRO analyses independently. All authors had full access to the data and accept responsibility to submit for publication.

## Data sharing statement

Raw antimicrobial susceptibility data extracted from the included studies are available in Supplementary Tables 2 and 3. The WISCA model specifications are available in Supplementary Table 4.

## Declaration of interests

Paul Turner is Co-PI for the ACORN2 AMR surveillance network project and was supported to attend the AMR diagnostic initiative meeting in Geneva in July 2023. PCMW is the recipient of an NHMRC grant focusing on antimicrobial resistance in children in Australia and the Southeast Asia/Pacific region, and was supported by ECCMID to attend the ASM/ECCMID conference on drug development to meet the challenge of antimicrobial resistance in October 2022.
