# Supplementary material for: Coverage gaps in empiric antibiotic regimens used to treat serious bacterial infections in neonates and children in Southeast Asia and the Pacific
Source: Lancet Reg Health Southeast Asia. 2023 Oct 31;22:100291. doi: 10.1016/j.lansea.2023.100291 (PMC10934317; doi:10.1016/j.lansea.2023.100291)
Supplement: Supplementary Table 1 [file mmc1.docx]

# Supplementary Table 1: Search Strategy

| Databases searched | PubMed, Cochrane, Embase, Global Health (plus a grey literature search). |
| --- | --- |
| Pre-specified inclusion criteria | 1. Research pertaining to bacterial infections: incidence, prevalence, aetiology, clinical infections 2. Isolates from sterile sites only, plus urine (if pertaining to clinical urinary tract infections) and stool (if pertaining to *Shigella* or *Salmonella*spp. clinical infections) 3. Specified paediatric data (age up to and including 18 years) 4. Antimicrobial testing methods documented and in line with CLSI/EUCAST recommendations 5. Published within the last 10 years 6. Data pertaining to bacterial GLASS 2020 organisms: *Escherichia coli, Klebsiella pneumoniae, Acinetobacter*spp., *Staphylococcus aureus, Streptococcus pneumoniae, Salmonella* spp., *Shigella*spp. *Neisseria gonorrhoea, Pseudomonas aeruginosa;* PLUS other bacterial pathogens particularly relevant in children: *Streptococcus agalactiae, Streptococcus pyogenes, Haemophilus influenzae, Neisseria meningitidis.* |
| Pre-specified exclusion criteria | 1. Data aggregated with adult data 2. Data aggregated with other regions outside the pre-defined geographic area 3. Isolates pertaining to carriage or colonisation studies 4. Small retrospective case series where n<10 5. Literature focussed on high-risk populations only (children LWHIV, profoundly immunosuppressed populations, children with SAM) 6. Poorly defined laboratory procedures, as defined by the MICRO framework |
| Limits | - January 1^st^ 2010 – March 14 2021 - Humans only |
| WPRO regional data: Included countries (excluding World Bank-defined high-income countries) | Cambodia, China, Laos PDR, Malaysia, Micronesia, Mongolia, PNG, Philippines, Solomon Islands, Samoa, Tonga, Vanuatu, Vietnam, Cambodia, Fiji, Kiribati |
| WPRO regional data: Search terms | (((antimicrobial[Text Word] OR antibiotic, resistan*[Text Word]) AND (antimicrobial[Text Word] OR antibiotic, susceptib*[Text Word] OR sensitiv*[Text Word])) AND (pediatr*[Text Word] OR paediatr*[Text Word] OR child*[Text Word] OR neonat*[Text Word] OR infant*[Text Word])) AND (cambodia[MeSH] OR china[MeSH] OR fiji[MeSH] OR laos[MeSH] OR malaysia[MeSH] OR micronesia[MeSH] OR mongolia[MeSH] OR papua new guinea[MeSH] OR philippines[MeSH] OR samoa[MeSH] OR tonga[MeSH] OR vanuatu[MeSH] OR vietnam[MeSH] OR cambodia[Text Word] OR china[Text Word] OR fiji[Text Word] OR  kiribati[Text Word] OR laos[Text Word] OR lao pdr[Text Word] OR lao people's democratic republic[Text Word] OR malaysia[Text Word] OR marshall islands[Text Word] OR micronesia[Text Word] OR mongolia[Text Word] OR papua new guinea[Text Word] OR philippines[Text Word] OR philipines[Text Word] OR phillipines[Text Word] OR phillippines[Text Word] OR samoa[Text Word] OR solomon island[Text Word] OR solomon islands[Text Word] OR tonga[Text Word] OR tuvalu [Text Word] OR vanuatu[Text Word] OR vietnam[Text Word] OR viet nam[Text Word] cambodian[Text Word] OR cambodians[Text Word] OR chinese[Text Word] OR fijian[Text Word] OR fijians[Text Word] OR lao[Text Word] OR laotian[Text Word] OR laotians[Text Word] OR malaysian[Text Word] OR malaysians[Text Word] OR marshallese[Text Word] OR micronesian[Text Word] OR micronesians[Text Word] OR mongolian[Text Word] OR mongolians[Text Word] OR mongol[Text Word] OR papua new guinean[Text Word] OR papua new guineans[Text Word] OR philippine[Text Word] OR philippines[Text Word] OR philipine[Text Word] OR philipines[Text Word] OR phillipine[Text Word] OR phillipines[Text Word] OR phillippine[Text Word] OR phillippines[Text Word] OR filipino[Text Word] OR filipinos[Text Word] OR filipina[Text Word] OR filipinas[Text Word] OR samoan[Text Word] OR samoans[Text Word] OR solomon islander[Text Word] OR solomon islanders[Text Word] OR tongan[Text Word] OR tongans[Text Word] OR tuvaluan[Text Word] OR tuvaluans[Text Word] OR vanuatu[Text Word] OR vanuatuan[Text Word] OR vanuatuans[Text Word] OR Vietnamese [Text Word]) |
| SEARO regional data: Included countries (excluding World Bank-defined high-income countries) | Bangladesh, Bhutan, DPR (North) Korea, India, Indonesia, Maldives, Myanmar, Nepal, Sri Lanka, Thailand, Timor-Leste |
| SEARO regional data: Search terms | (((antimicrobial or antibiotic, resistan*) and (susceptib* or sensitiv*)) OR ((antibacterial or anti microbial or bacteria or microbial) adj4 resist*)) AND (Bangla* or Bhutan* or DPRK* or North Korea* or India* or Indonesia* or Maldiv* or Myanm* or Nepal* or Sri Lanka* or Thai* or Timor*) AND (pediatr* or paediatr* or child* or neonat* or infant*))) |
