# Supplementary material for: Coverage gaps in empiric antibiotic regimens used to treat serious bacterial infections in neonates and children in Southeast Asia and the Pacific
Source: Lancet Reg Health Southeast Asia. 2023 Oct 31;22:100291. doi: 10.1016/j.lansea.2023.100291 (PMC10934317; doi:10.1016/j.lansea.2023.100291)
Supplement: Supplementary Table 2 [file mmc2.docx]

| **First author** | **Year of Pub- lication** | **Title** | **Study design** | **Prospective vs Retrospective** | **Country** | **Setting** | **Age range** | **n** | **Denominator (if given)** | **CA vs HA** | **Specimen type** | **Target organism(s)** | **Total # bacterial isolates from relevant cultures** | **Specimen processing / target organism identification methodology** | **Susceptibility guidance (CLSI, EUCAST) etc where defined** | **Study Limitations** | **GRADE (A-D)** | **GRADE level rationale** | **MICRO (A-E)** | **MICRO level rationale** |
| --- | --- | --- | --- | --- | --- | --- | --- | --- | --- | --- | --- | --- | --- | --- | --- | --- | --- | --- | --- | --- |
| **Adhikari** | 2014 | Bacteriological profile and associated risk factors of neonatal sepsis in Paropakar Maternity and Women's Hospital Thapathali, Kathmandu. | Cross sectional | Prospective | Nepal | Urban tertiary | 0 | 94  total  40  followi ng exclusi on of CoNS & Pseud spp. (as not defined as aerugin osa) | 452 neonates with suspected sepsis | N/A | BC | E coli, S aureus, K pneumoniae, Pseudomona s spp (not defined - therefore excluded). Staph epi (excluded) | 39 | Blood innoculated into various broths, Gram staining, colony characteristics, biochemical properties, and slide agglutination where appropriate | Not defined | No delineation of CA vs HA Includes CoNS (without delineation of  clinical correlation) Limited AST reporting, no mention MDR  No strategy for duplicates | D | Small sample size of clinically relevant organisms Limited methodology description with non- standardized definition of sepsis Limited AST testing,  ?micro errors | D | No accreditation/EQA mentioned  Manual organism ID  Pseud reported CTX susceptible CLSI disc diffusion  No method for duplicates |

| **DENIS** | 2016 | Characterisatio n and antimicrobial resistance of sepsis pathogens in neonates born in tertiary care centres in Delhi, India: a cohort study | Cohort | Prospective | India | Urban tertiary | 0 | 88636  live births 13530  to NICU 4650  suspect sepsis 840  culture pos sepsis 200  mening itis | 4650 suspected sepsis | N/A | BC CSF | Acinetobacte r spp Klebsiella spp E.Coli Pseudomona s Enterobacter CoNS  S.Aureus Enterococcus GBS | 1040 | Not stated. Presumed automated | Not defined | ?Acinetobacter primary pathogen Included CoNS if clinical criteria met with only one positive culture EQA mentioned but no further detail re micro methods | B | Single high quality prospective study with descriptive methodology Recognises difficulty in definition of neonatal sepsis  Good sample size  Clinically relevant outcomes including CFR in culture positive sepsis with sensitive v resistant organisms Distinguishes EOS and LOS  Specific to NICU  population (2/3rd LBW and 50% preterm) Included CoNS if clinical criteria met EQA  mentioned but no further detail re micro methods | B | No accreditation details EQA participation confirmed  Organism ID and AST method NOT described |
| --- | --- | --- | --- | --- | --- | --- | --- | --- | --- | --- | --- | --- | --- | --- | --- | --- | --- | --- | --- | --- |

| **Annamali** | 2021 | Increasing Resistance to Reserve Antibiotics: The Experience of a Tertiary Level Neonatal Intensive Care Unit. | Cohort | Prospective | India | Urban tertiary | 0 | 898  suspect ed neonat al sepsis 107  positiv e culture s  82  after exclusi ons of CoNS | 898 | N/A | BC | Klebsiella pneumoniae CoNS  Acinetobacte r Staphylococc us aureus Enterococcus faecalis Pseudomona s aeruginosa E.Coli | 82 | Innoculated brain heart infusion broth. Incubated 37 degrees.  Subculture on blood, chocolate and MacConkey. Organisms identified with "standard biochemical tests" | Not defined | No strategy duplicates Ampicillin/sulbacta m and amikacin first line EOS, mero/vanc LOS | B | Prospective, good sample size, clinical outcome data and risk factors provided Clear definitions for sepsis, EOS/LOS,  MDR given  No strategy duplicates Specific to this unit/India: Ampicillin/sul bactam and amikacin first line EOS, mero/vanc LOS | C | No accreditation/EQA not mentioned  ?Automated v manual CLSI disc diffusion  No glaring micro errors, good range AST testing |
| --- | --- | --- | --- | --- | --- | --- | --- | --- | --- | --- | --- | --- | --- | --- | --- | --- | --- | --- | --- | --- |

| **Ansari** | 2014 | Childhood septicemia in Nepal: documenting the bacterial etiology and its susceptibility to antibiotics. | Cross sectional | Prospective | Nepal | Urban tertiary | 0.5-  15 | 104  (172  minus 68 CoNS) | 1630 children suspeted sepsis | N/A | BC | CoNS  Staph aureus Enterococcus spp Acinetobacte r spp Pseudomona s aeruginosa Citrobacter spp Enterobacter spp Klebsiella pneumoniae Eschericia coli Salmonella spp | 104 | Brain heart infusion broth 37 degree incubation. Bottles examined for turbidity, haemolysis and pellicle formation then subcultures made onto sheep blood, chocolate and MacConkey agar after overnight incubation. Bottles observed and subcultured each day.  Conventional biochemical tests and standard microbiologica l methods used (reference given) | Not defined | Inclusion criteria not well defined (ie inaptient/outpatie nt/CA v HA)  No clinical outcome data | C | Good sample size, likely prospective although not explicitly stated  Inclusion criteria not well defined Duplicates not accounted for. No note of previous antibiotic exposure No clinical  outcome data CoNs and all acinetobacter spp of dubious significance as most common organisms in non-neonatal age group | C | No accreditation/EQA not mentioned  ?Automated v manual CLSI disc diffusion Good range AST testing  Reports cefoxitin and multiple betalactams for S.Aureus |
| --- | --- | --- | --- | --- | --- | --- | --- | --- | --- | --- | --- | --- | --- | --- | --- | --- | --- | --- | --- | --- |

| **Balaji** | 2015 | Pneumococcal serotypes associated with invasive disease in under five children in India & implications for vaccine policy. | Case control | Prospective | India | Urban tertiary | 0.5-5 | 114 | Nil | N/A | Blood, CSF, pleural fluid | S.pneumonia e | 114 | BacT/ALERT for 7 days. Sheep blood agar then alpha haemolytic colonies sub- cultured onto 10% sheep agar and characterised with bile soluble test and optochin susceptibility test. | Not defined | No clinical information, no denominator provided, no strategy for duplicates, no mention previous antibiotic use | D | Large number isolates for single organisms, automated microbiology  No clinical data, no strategy for duplicates, no mention previous antibiotic use | D | No accreditation/EQA not mentioned  Use of BacT/ALERT and Vitek although only 50%  Limited but ?appropriate range AST testing although no R/I/S and no actual numbers, just %'s |
| --- | --- | --- | --- | --- | --- | --- | --- | --- | --- | --- | --- | --- | --- | --- | --- | --- | --- | --- | --- | --- |

| **Banerjee** | 2016 | Long-term outbreak of Klebsiella pneumoniae & third generation cephalosporin use in a neonatal intensive care unit in north India. | Case series | Retrospective | India | Urban tertiary | 0 | 43 | Nil | N/A | Blood | Klebsiella pneumoniae | 43 | "Standard protocol" and "extensive biochemical tests" with reference to Mackie and Macartney textbook | Not defined | Case series, no strategy for duplicates | D | Case series, poor descripiton microbiology  Good clinical information, includes previous antibiotic exposure (specifically 3GC),  differentiate EOS/LOS and duration of stay in hospital | D | No accreditation/EQA not mentioned  Poor description of organism identification  Ampicillin tested for K.pneumo Disc diffusion |
| --- | --- | --- | --- | --- | --- | --- | --- | --- | --- | --- | --- | --- | --- | --- | --- | --- | --- | --- | --- | --- |
| **Bandyopad hyay** | 2018 | Distribution, antimicrobial resistance and predictors of mortality in neonatal sepsis. | Cohort | Retrospective | India | Urban tertiary | 0 | 139  (exclud ing 42 CoNS  and 2 pseudo monas spp) | Nil | N/A | Blood | Klebsiella spp E.Coli Acinetobacte r spp Pseudomona s spp Cirobacter spp  CoNs Enterococcus spp  S.aureus | 139 | Not described | Not defined | Retrospective  No description of micro methodology | D | Moderate sample size Good clinical outcome data including CFR of resistant v non resistant organisms  Poor micro quality Retrospective No denominator | E | No accreditation/EQA not mentioned  No description of organism identification  No AST guideline mentioned |

| **Batty** | 2020 | The spread of chloramphenic ol-resistant Neisseria meningitidis in Southeast Asia. | Case series | Retrospective | Thailand | Urban tertiary | 0-18 | 11 | Nil | N/A | Blood, CSF | N.meningitidi s | 11 | MALDI-TOF | CLSI (MICs and R/I/S reported) | Small sample No denominator | D | Small retrospective case series Minimal clinical data  Reliable micro Duplicates accounted for | C | No accreditation/EQA not mentioned  Use of MALDITOF for organism identification  AST by Etest |
| --- | --- | --- | --- | --- | --- | --- | --- | --- | --- | --- | --- | --- | --- | --- | --- | --- | --- | --- | --- | --- |
| **Britto** | 2018 | Laboratory and molecular surveillance of paediatric typhoidal Salmonella in Nepal: Antimicrobial resistance and implications for vaccine policy | Cohort | Prospective | Nepal | Urban tertiary | 0-14 | 264  (from supple mentar y) | Nil | CA | Blood | Salmonella spp | 264 | Batec incubator 37  degrees for 5 days.  Innoculated onto MacConkey then suspected colonies subjected to standard biochemical tests | Not defined | Minimal clinical information  No strategy for duplicates  No mention previous antibiotic use  Denominator for inpatients but not outpatients | C | High quality, prospective, good sample size, appropriate denominator  Minimal clinical information No strategy for duplicates No mention previous antibiotic use Denominator for inpatients but not outpatients | D | No accreditation/EQA not mentioned  Organism identification not well described  AST data presented as percentages only  No macrolide resistance presented |

| **Britto** | 2020 | Persistent circulation of a fluoroquinolon e-resistant Salmonella enterica Typhi clone in the Indian subcontinent. | Cross sectional | Prospective | India | Urban tertiary | 0-18 | 37 | Nil | N/A | Blood | Salmonella spp | 37 | Biochemical and serological | CLSI inhibition zone >31mm and MIC <0.06 for  cipro and <0.12 for ofloxacin on VITEK | No clinical information, no denominator provided, no strategy for duplicates, no mention previous antibiotic use | D | High quality, prospective, delineates adult/childre n  No child denominator Minimal clinical information No strategy for duplicates No mention previous antibiotic use | C | No accreditation/EQA AST by Vitek |
| --- | --- | --- | --- | --- | --- | --- | --- | --- | --- | --- | --- | --- | --- | --- | --- | --- | --- | --- | --- | --- |
| **Chakkarap ani** | 2014 | Pattern and antimicrobial susceptibility of carbapenem resistant organisms in tertiary care neonatal intensive care unit, India. | Cohort | Retrospective | India | Urban tertiary | 0 | 134 | 2720 | N/A | Blood | Acinetobacte r spp Klebsiella spp E.Coli Serratia marcescens | 15 | BACTEC9120  automated or brain heart agar with biochemical tests | Not defined | Retrospective | D | Retrospective  , small sample size, no strategy for duplicates,  ?colistin susceptibility testing, high rates culture positivity  Pragmatic inclusion criteria, has mortality information | D | No accrediation/EQA Mixture BATEC/biochemical tests  Unusual AST testing  AST data as percentages only  ?Colistin susceptibility test |

| **Chelliah** | 2014 | Isolation of MRSA, ESBL  and AmpC -  $\beta$ - lactamases from Neonatal Sepsis at a Tertiary Care Hospital. | Cross sectional | Prospective | India | Urban tertiary | 0 | 98 (110  minus 12 CoNS) | 182 | N/A | Blood | Klebsiella pneumoniae E.Coli S.aureus E.Faecalis Klebsiella spp P.aeruginosa | 98 | Brain heart infusion broth with "standard microbiologica l procedure" | Cefoxitin disc for MRSA  ESBL phenotypic combined disc test + 3 times doubling dilution reduction MIC of 3GC in presence of Clavulanic acid considered ESBL production | Very high culture positivity rate, minimal clinical information, no strategy for duplicates | D | Very high culture positivity rate, minimal clinical information, no strategy for duplicates, poor micro reporting  Moderate sample size, prospective | E | No accreditation/EQA mentioned  Manual organism ID CLSI disc diffusion  Data not comprehensively reported in table ESBL/MRSA reporting appropriate |
| --- | --- | --- | --- | --- | --- | --- | --- | --- | --- | --- | --- | --- | --- | --- | --- | --- | --- | --- | --- | --- |

| **Chung** | 2015 | A high- resolution genomic analysis of multidrug- resistant hospital outbreaks of Klebsiella pneumoniae | Case series | Retrospective | Nepal | Urban tertiary | 0-18 | 29 | Nil | N/A | BC | Klebsiella pneumoniae | 29 | BACTEC then biochemical tests including API20E | Not defined | Outbreak/case series, retrospective | D | Outbreak/cas e series, retrospective, no duplicate strategy  High quality analysis, complex genomics, high mortality reported | D | No accreditation/EQA mentioned  Use of control disc explicitly stated  Reports AMX S for K.pneumo Automated bacteriology |
| --- | --- | --- | --- | --- | --- | --- | --- | --- | --- | --- | --- | --- | --- | --- | --- | --- | --- | --- | --- | --- |
| **Datta** | 2014 | A five-year experience of carbapenem resistance in Enterobacteria ceae causing neonatal septicaemia: predominance of NDM-1. | Cohort | Retrospective | India | Urban tertiary | 0 | 285 | 1985 | N/A | BC | E.Coli K.pneumonia e | 95 | ID 32 E Kit | ESBL, Amp C, KPC,  MBL phenotypes evaluated using cephalosporin/cla v disc, cefoxitin/boronic acid, mero/boronic acid | Retrospective, not all organisms reported on | C | Good sample size with denominator included, clinical information, strategy for duplicates, quality micro and molecular methods  Retrospective  , not all organisms reported on | C | No accreditation/EQA mentioned  Good description of micro methods  Strategy for duplicates mentioned  AST in proportions only |

| **Devi** | 2018 | Extended- spectrum beta- lactamase & carbapenemas e-producing Gram-negative bacilli in neonates from a tertiary care centre in Dibrugarh, Assam, India. | Cohort | Prospective | India | Urban tertiary | 0 | 21 | 303 | N/A | CSF | Acinetobacte r spp Klebsiella spp Pseudomona s spp  E.Coli | 21 | Not described | Phenotypic/Geno typic ESBL/MBL identification | Small sample size, gram negative organisms only, no clinical informaion | D | Small sample size, gram negative organisms only, no clinical information  Prospective, genotypic resistance reported | E | No accreditation/EQA mentioned  Some description of micro methods  Including clear ESBL/MBL detection method description |
| --- | --- | --- | --- | --- | --- | --- | --- | --- | --- | --- | --- | --- | --- | --- | --- | --- | --- | --- | --- | --- |

| **Dey** | 2020 | Is Superbug imminent? findings of a retrospective study in Bangladesh. | Cohort | Retrospective | Banglad esh | Urban tertiary | 0 | 124 | 559 | N/A | BC | Acinetobacte r spp Klebsiella spp E.Coli | 124 | Trypticase soya broth then standard techniques | Not defined | Retrospective, poor micro description, organisms by genus only, 90% LOS | D | Retrospective  , poor micro description, organisms by genus only, no strategy for duplicates  Large sample size, good clinical information provided with clear inclusion criteria/deno minator | E | No accreditation/EQA mentioned  Organism ID/AST not mentioned Genus not spp  No strategy for duplicates |
| --- | --- | --- | --- | --- | --- | --- | --- | --- | --- | --- | --- | --- | --- | --- | --- | --- | --- | --- | --- | --- |

| **Dhanawad e** | 2015 | Pattern and antimicrobial susceptibility of neonatal sepsis at a tertiary care center in western India. | Cohort | Retrospective | India | Urban tertiary | 0 | 121  (110  after removi ng CoNS) | 620 | N/A | BC | Acinetobacte r spp Klebsiella spp E.Coli S.aureus CoNS  Enterococcus | 110 | Brain heart infusion broth and subcultures on blood/MacCon key. "Conventional methods" | Not defined | Retrospective, no strategy for duplicates, use of piptaz/amikacin as first line | C | Retrospective  , no strategy for duplicates, use of piptaz/amikac in as first line  Large sample size, good clinical information provided including mortality with clear inclusion criteria/deno minator | D | No accreditation/EQA mentioned  Organism ID not described CLSI disc diffusion  No strategy for duplicates Ampicillin tested for kleb |
| --- | --- | --- | --- | --- | --- | --- | --- | --- | --- | --- | --- | --- | --- | --- | --- | --- | --- | --- | --- | --- |

| **Gajul** | 2015 | Klebsiella Pneumoniae in Septicemic Neonates with Special Reference to Extended Spectrum  $\beta$- lactamase, AmpC, Metallo  $\beta$- lactamase Production and Multiple Drug Resistance in Tertiary Care Hospital. | Cohort | Retrospective | India | Urban tertiary | 0 | 24 | 114 | N/A | BC | Klebsiella pneumoniae | 24 | BacT/ALERT for 7 days then subculture on MacConkeys organism ID "standard microbiologica l methods" | ESBL, Amp C, KPC,  MBL phenotypes evaluated using cephalosporin/cla v disc, cefoxitin/boronic acid, mero/boronic acid | Retrospective, no clinical information, inclusion criteria not mentioned, no strategy for duplicates, very high culture positivity rates reported | D | Retrospective  , no clinical information, inclusion criteria not mentioned, no strategy for duplicates, very high culture positivity rates reported | D | No accreditation/EQA mentioned  Organism ID "standard" CLSI disc diffusion  No strategy for duplicates Ampicillin tested for kleb |
| --- | --- | --- | --- | --- | --- | --- | --- | --- | --- | --- | --- | --- | --- | --- | --- | --- | --- | --- | --- | --- |

| **Gyawali** | 2013 | Bacteriological profile and antibiogram of neonatal septicemia | Cohort | Prospective | Nepal | Urban tertiary | 0 | 227  (238  minus 11 CoNS) | 1572 | N/A | BC | S.aureus CoNS  Klebsiella spp Enteobacter spp  E.Coli Citrobacter Acinetobacte r spp Pseudomona s spp | 227 | Brain hear infusion broth then standard microbiologica l method | Not defined | No demographic or outcome information, no strategy for duplicates, AST data for Enterobacterales not individual | D | Large sample size, prospective, appropriate inclusion/den ominator  No demographic or outcome information, no strategy for duplicates, AST data for Enterobacter ales not individual | E | No accreditation/EQA mentioned  "Standard microbiological methods"  CLSI disc diffusion  No strategy for duplicates Only breaks down AST for all Gm negs/pos |
| --- | --- | --- | --- | --- | --- | --- | --- | --- | --- | --- | --- | --- | --- | --- | --- | --- | --- | --- | --- | --- |

| **Jajoo** | 2018 | Alarming rates of antimicrobial resistance and fungal sepsis in outborn neonates in North India. | Cohort | Prospective | India | Urban tertiary | 0 | 240  (401  minus fungal, "other"  ) CoNS  include d as defined | 2588 | Y | BC | Klebsiella pneumoniae Acinetobacte r baumanii E.Coli E.cloacae S.aureus E.faecium CoNS | 203 | BacT/ALERT->  Vitek 2 | Mentioned S/I/R but then not reported | Single centre, outborn neonates, high rates fungal sepsis (7%) | A | Single centre, outborn neonates, high rates fungal sepsis (7%)  Large sample size, high quality prospective study, well described strict inclusion criteria, no dropouts, demographic/ clinical outcome data presented, attempt to deal with duplicates, previous Abx use mentioned, defines HAI/CA | B | No accreditation EQA participation  Vitek 2 used, internal controls Rigorous and accurate AST reporting |
| --- | --- | --- | --- | --- | --- | --- | --- | --- | --- | --- | --- | --- | --- | --- | --- | --- | --- | --- | --- | --- |

| **Jatsho** | 2020 | Clinical and bacteriological profile of neonatal sepsis: a prospective hospital-based study. | Cross sectional | Prospective | Bhutan | Urban tertiary | 0 | 33 (44  minus CoNS) | 314 | N/A | BC | Klebsiella pneumoniae Acinetobacte r spp  E.Coli  K oxytoca Citrobacter spp  S pneumo Enterococcus spp | 33 | BacT/ALERT for 5 days at 37 degrees. Org ID not described | I= resistant (CLSI) | Single centre, no clinical outcome data | B | Single centre, no clinical outcome data  Moderate sample size, high quality prospective study, well described inclusion criteria, demographic/ RF data | D | No accreditation  Automated culture method but no description of org ID or AST Genus only acinetobacter/citrobacter/enter ococcus |
| --- | --- | --- | --- | --- | --- | --- | --- | --- | --- | --- | --- | --- | --- | --- | --- | --- | --- | --- | --- | --- |

| **Jayaraman** | 2018 | Burden of bacterial meningitis in India: preliminary data from a hospital based sentinel surveillance network. | Cross sectional | Prospective | India | Urban tertiary | 0.5-5 | 257 | 3104 | N/A | CSF | S  pneumoniae H influenzae N  meningitidis | 257 | Microscopy, culure and latex agglutination test | Not defined | Small number isolates ultimately AST tested, no clinical outcome data, no AST testing for H.flu/N.menin, 29/213 pneumo cases able to have AST testing done | C | Prospective, large multi- centre, clear and pragmatic inclusion criteria  Small number isolates ultimately AST tested, no clinical outcome data, no AST testing for H.flu/N.meni n, 29/213  pneumo cases able to have AST testing done | C | No accreditation EQA participation  No clear detail on organism ID Vitek 2 used for AST  AST only for s.pneumo |
| --- | --- | --- | --- | --- | --- | --- | --- | --- | --- | --- | --- | --- | --- | --- | --- | --- | --- | --- | --- | --- |

| **Khan** | 2014 | Epidemiology and drug resistance profile of acute bacterial meningitis in children in Northern India: A university hospital perspective | Cohort | Prospective | India | Urban tertiary | 0-15 | 79 | 150 | N/A | CSF | S  pneumoniae E coli  N  meningitidis K  pneumoniae S aureus  P aeruginosa S agalactiae HIB  C koseri L  monocytogen es | 79 | ID based on colony morphology, culture characterstics and biochemical reactions | Not defined ESBL/Amp C screening described | Small numbers of isolates for individual organisms for AST testing, no duplicate strategy, culture negative meningitis common, no penicillin MIC for s pneumo | B | Prospective, moderate sample size for meningitis, clear description of inclusion criteria  Small numbers of isolates for individual organisms for AST testing, no duplicate strategy, culture negative meningitis common, no penicillin MICs for s pneumo | D | No accreditation/EQA mentioned  Organism ID by colony morph and biochemical  CLSI disc diffusion AST with internal controls  K pneumo tested amp, no pen MICs, pseud tested for CRO |
| --- | --- | --- | --- | --- | --- | --- | --- | --- | --- | --- | --- | --- | --- | --- | --- | --- | --- | --- | --- | --- |

| **Khanal** | 2014 | Bacteriological profile of neonatal sepsis in a tertiary level hospital of Nepal. | Cross sectional | Prospective | Nepal | Urban tertiary | 0 | 19 (69  minus 50 CoNS) | 340 | N/A | BC | E coli Klebsiella spp S aureus  S epidermidis S  saprophyticu s  E faecalis | 19 | Characteristic apparance, gram stain and biochemical reaction | Not defined | Small number of isolates, predominant CoNS, no inclusion criteria/demograph ic info/outcome data | D | Prospective  Small number of isolates, predominant CoNS, no inclusion criteria/demo graphic info/outcome data | D | No accreditation/EQA mentioned  Organism ID by colony morph and biochemical  CLSI disc diffusion AST No MDR definition  K pneumo tested amp |
| --- | --- | --- | --- | --- | --- | --- | --- | --- | --- | --- | --- | --- | --- | --- | --- | --- | --- | --- | --- | --- |
| **Kumar** | 2014 | Risk factors for carbapenem- resistant Acinetobacter baumanii blood stream infections in a neonatal intensive care unit, Delhi, India. | Cohort | Retrospective | India | Urban tertiary | 0 | 65 | 474 | Y | BC | A baumanii | 65 | BacT/Alert-> Vitek 2 | Resistance to carbapenem MIC>16mcg/mL | Retrospective, single centre, single organism study | C | Retrospective  , single centre, single organism study  High quality study with demographic information and clinically releavant outcomes, including information on prior abx use and LOS | C | No accreditation/EQA mentioned  Organism ID by vitek CLSI disc diffusion AST No strategy duplicates |

| **Mehar** | 2013 | Neonatal sepsis in a tertiary care center in central India: Microbiological profile, antimicrobial sensitivity pattern and outcome | Cohort | Retrospective | India | Urban tertiary | 0 | 45 (58  minus 12  GPCs, 1  steno) | 285 | Inborn v outborn | BC | Klebsiella spp E Coli  P aeruginosa Enterobacter spp Acinetobacte r spp Pantoea dispersa  S mercescans S maltophila S aureus  S  haemolyticus CoNS  Streptococcu s | 45 | BacT/Alert-> Vitek 2 | Not defined | Retrospective, single centre, high mortality rate, no AMR clinical outcome data, no duplicates strategy | C | Moderate sample, clear inclusion criteria, polymicrobial excluded  Retrospective  , single centre, small sample, high mortality rate, no AMR clinical outcome data, no duplicates strategy | D | No accreditation/EQA mentioned  Organism ID/AST by Vitek No stratgy duplicates Ampicillin reported for kleb |
| --- | --- | --- | --- | --- | --- | --- | --- | --- | --- | --- | --- | --- | --- | --- | --- | --- | --- | --- | --- | --- |

| **Mitra** | 2019 | Evaluation of co-transfer of plasmid- mediated fluoroquinolon e resistance genes and bla (NDM) gene in Enterobacteria ceae causing neonatal septicaemia. | Cohort | Not clear | India | Urban tertiary | 0 | 73 | None | N/A | BC | E Coli  K pneumo E cloacae  E aerogenes | 73 | Vitek2 | Not defined | High rates of resistance, Limited methods/informati on about collection of samples, no clinical information, no strategy for duplicates | C | High quality molecular testing  Limited methods/info rmation about collection of samples, no clinical information, no strategy for duplicates | C | No accreditation/EQA mentioned  Organism ID Vitek AST disc diffusion  No stratgy duplicates |
| --- | --- | --- | --- | --- | --- | --- | --- | --- | --- | --- | --- | --- | --- | --- | --- | --- | --- | --- | --- | --- |

| **Muley** | 2015 | Bacteriological profile of neonatal septicemia in a tertiary care hospital from Western India | Cohort | Not clear | India | Urban tertiary | 0 | 41 (48  minus 3 CoNS  and 4 pseud spp) | 180 | N/A | BC | K  pneumoniae E Coli Acinetobacte r spp  S aureus | 41 | Tryptose phosphate broth and "standard bacteriological procedure) | Not defined | No dates, no inclusion criteria/demograph ic information, poor micro methodology, high  % sepsis, no strategy duplicates | D | Moderate sample size  No dates, no inclusion criteria/demo graphic information, poor micro methodology, high % sepsis, no strategy duplicates | E | No accreditation/EQA mentioned  Organism ID "standard" AST disc diffusion  Orgs not reported to spp level No strategy for duplicates  No dates or study |
| --- | --- | --- | --- | --- | --- | --- | --- | --- | --- | --- | --- | --- | --- | --- | --- | --- | --- | --- | --- | --- |

| **Mya** | 2017 | In-vitro antimicrobial susceptibility pattern of blood culture isolates from childhood invasive bacterial infections. | Cross sectional | Prospective | Myanm ar | Urban tertiary | 0-18 | 27 | 140 | CA | BC | Pseudomona s spp  E coli Citrobactr freundii Enterobacter spp  N  meningitidis S aureus  L  monocytogen es  S viridans | 27 | Tryptic soy broth at 37  degrees for 7 days and examined macroscopicall  y. Subculture onto blood, chocolate, macconkey, mannitol salt. Morphological and biochemicalID | Yes CLSI S/I/R | Single centre, sample size, poorly described inclusion criteria, no strategy for duplicates/previou s antimicrobials mentioned | C | Single centre, sample size, poorly described inclusion criteria, no strategy for duplicates/pr evious antimicrobial s mentioned  Prospective | E | No accreditation/EQA mentioned  Organism ID biochemical AST disc diffusion  Incomplete AST data presented Unusual susceptibility pattern for S aureus  No duplicate strategy  Clear AMR/MDR definitions |
| --- | --- | --- | --- | --- | --- | --- | --- | --- | --- | --- | --- | --- | --- | --- | --- | --- | --- | --- | --- | --- |

| **Gopi** | 2016 | Epidemiological characterisatio n of Streptococcus pneumoniae from India using multilocus sequence typing | Cohort | Prospective | India | Urban tertiary | <5yo | 37  (exclud ing "fluid" from pneum onia and empye ma) | None | CA | Sterile body fluids (blood, CSF) | Streptococcu s pneumoniae | 37 | S. pneumoniae identified using standard methods such as optochin susceptibility test and bile solubility test. Isolate serotypes determined using co- agglutination technique with Neufeld antisera and sequential conventional multiplex polymerase chain reaction (SMPCR) | CLSI | Small sample size, inclusion of non- sterile body fluids ("Fluid" from pneumonia/empye ma), exclusion of NP carriage isolates, exclusion of non-invasive isolates, MDR not defined | C | Small sample size, prospective cohort study. Clinical characteristic s, serotype, sequence type and antimicrobial resistance profile well described | C | No accreditation details provided, EQA participation not confirmed, organism details very well described, small range of antimicrobials tested, VITEK version stated (VITEK 2), and CLSI version stated (2013), DNA extraction, Multilocus sequence typing, and DNA editing performed (in addition to AST) |
| --- | --- | --- | --- | --- | --- | --- | --- | --- | --- | --- | --- | --- | --- | --- | --- | --- | --- | --- | --- | --- |

| **Khan** | 2015 | Klebsiella pneumoniae Outbreak in Paediatric Ward: Detection and Prevention | Cohort | Prospective | India | Urban tertiary | Not repor ted | 90 | Nil | HA | Blood | Klebsiella pneumoniae | 90 | Samples were received for blood culture in brain heart infusion broth. Repeated subcultures were done on 5% sheep Blood agar and Mac-Conkeys agar after 24  hours, 48  hours and 7 days of incubation at 37°C. Cultures showing growth were identified by standard biochemical procedures | CLSI | Minimal clinical information, moderte sample size, ages of sample population not provided, no strategy for duplicates mentioned,  no mention previous antibiotic use, no clear denominator provided, presumed prospective | D | Prospective cohort study, moderate sample size, minimal clinical information, ages of sample population not provided, no strategy for duplicates mentioned, no mention previous antibiotic use, no clear denominator provided, presumed prospective | C | No accreditation details provided, EQA participation not confirmed, broad range of antibiotics |
| --- | --- | --- | --- | --- | --- | --- | --- | --- | --- | --- | --- | --- | --- | --- | --- | --- | --- | --- | --- | --- |

| **Khanam** | 2015 | Typhoid Fever in Young Children in Bangladesh: Clinical Findings, Antibiotic Susceptibility Pattern and Immune Responses | Cohort | Prospective | Banglad esh | Urban (field sites, and the Dhaka hospital  ) | 1 -  59yo (how ever, data for 1 -  17 yo extra cted) | 56  (paedia tric patient s, 1 -  17yo) | Nil | CA | Blood | S. Typhi | 56 | Blood was cultured using a BacT/Alert automated system, and positive cultures characterised using standard bacteriological procedures. | CLSI | No strategy for duplicates mentioned, small sample size, presumed prospective, no time period for data collection given | D | Presumed prospective cohort study, small sample size, no strategy for duplicates mentioned, no clear denominator provided, no time period for data collection given | C | Accreditation details not provided, EQA participation not confirmed, organism ID and AST methodology completely described |
| --- | --- | --- | --- | --- | --- | --- | --- | --- | --- | --- | --- | --- | --- | --- | --- | --- | --- | --- | --- | --- |

| **Mahich** | 2020 | Acinetobacter Sepsis Among Out-born Neonates Admitted to Neonatal Unit in Pediatric Emergency of a Tertiary Care Hospital in North India | Cohort | Prospective | India | Urban tertiary | 0 | 43 | 406 | Both | Blood | Acinetobacte r spp. | 43 | Blood collected into Pediatric BACTEC blood culture bottles, incubated at 37 °C, and cultured using the BACTEC systems. If flagged positive, gram staining was performed and subculture was done on appropriate media. Bottles were incubated in the system for 5 d and identification of organisms was done by Matrix assisted laser desorption ionization. | CLSI | AST reported for Acinetobacter spp. (not A. baumanii versus A. junii), the same antimicrobials not tested on all specimens, single centre study, small sample size, no long-term follow- up, nil other markers of sepsis (CRP etc), large drop out rate (63%) secondary to: death, and leaving against medical advice, high proportion of pre- term neonates (46.5%, n = 20). | C | Small sample size, subgroup analysis of a a larger prospective study, a great deal of clinical information provided, no strategy for duplicates mentioned, previous antibiotic use mentioned, perinatal risk factors explored, final outcomes well described, large drop out rate (63%)  secondary to: death, and left against medical advice | D | Accreditation details not provided, EQA participation not confirmed, antimicrobial susceptibility not provided per strain (e.g. A. baumanii versus A. junii), the same antimicrobials not tested on all specimens, broad range of antibiotics |
| --- | --- | --- | --- | --- | --- | --- | --- | --- | --- | --- | --- | --- | --- | --- | --- | --- | --- | --- | --- | --- |

| **Nagaraj** | 2017 | Streptococcus pneumoniae serotype prevalence and antibiotic resistance among young children with invasive pneumococcal disease: experience from a tertiary care center in South India. | Observatio nal cohort | Prospective | India | Urban tertiary | <5yo | 14  (unable to disaggr egate blood and CSF  only) | 171 | CA | Blood, cerebrospinal fluid (CSF), and pleural fluid | Streptococcu s pneumoniae | 14 | Specimens (blood, CSF, and pleural fluid) collected in a standard blood culture bottle (Bactec Peds Plus) and incubated at 35 °C in  BACTEC 9240  instrument. Positive specimens then plated on to trypticase soy agar (Himedia) supplemented with 5% sheep blood, chocolate agar plates and incubated in a candle jar.  Suspected S. pneumoniae colonies were identified by α-hemolysis,  typical colonial morphology, presence of diplococci on Gram stain, optochin sensitivity (Taxo P disks) and confirmed by testing for bile solubility. All isolates were confirmed again by multiplex PCR, serogrouped/t yped with Quellung antisera. | CLSI (susceptible, intermediate, or resistant) | Small sample size,  S. pneumoniae isolation rate only 8.2% among children with suspected IPD, data is from a  single hospital, data not able to be disaggregated based on blood/CSF/pleural fluid | C | Prospective, observational study, strategy for duplicates well defined, small sample size, paediatric only population, clinical syndromes well described, prior antibiotic treatment described | C | Accreditation details not provided, EQA participation not confirmed, organism ID and AST methodology completely described, organism isolated by routine laboratory methods confirmed by the reference laboratory, only one isolate  per patient was included, further serotyping of organisms performed, no ID or AST errors detected |
| --- | --- | --- | --- | --- | --- | --- | --- | --- | --- | --- | --- | --- | --- | --- | --- | --- | --- | --- | --- | --- |

| **Punpanich** | 2012 | Risk factors for carbapenem non- susceptibility and mortality in Acinetobacter baumannii bacteremia in children. | Case series | Retrospective | Thailand | Urban tertiary | 0 -  18yo | 180 | 74 955 | Both | Blood | Acinetobacte r baumannii | 180 | A. baumannii were isolated and identified from clinical specimens by standard microbiologica l methods | CLSI (susceptible, intermediate, or resistant) | Single centre study, cases not disaggregated by age, total peripheral white blood cell count was in insensitive screening tool for bacteremia in children, described AST with multiple antimicrobials however data not given, nil strategy for duplicates described | D | Retrospective  , case series, moderate sample size, single centre study clinical outcomes well documented, baseline clinical and demographic characteristic s provided, leukocyte count provided, risk factors well delineated, described AST with multiple antimicrobial s however data not given, previous antibiotic use well described, nil strategy for duplicates described | D | Accreditation details not provided, EQA participation not confirmed, organism ID and AST methodology completely described for carbapenem alone, described AST with multiple antimicrobials however data not given, previous antibiotic use well described |
| --- | --- | --- | --- | --- | --- | --- | --- | --- | --- | --- | --- | --- | --- | --- | --- | --- | --- | --- | --- | --- |

| **Ravi** | 2013 | Circulating serotypes and trends in antibiotic resistance of invasive Streptococcus Pneumoniae from children under five in Bangalore | Cohort | Prospective | India | Urban tertiary | <=5y  o | 40 | Nil | CA | Blood, CSF and pleural fluid (note: AST not disaggregate d by specimen type) | Streptococcu s Pneumoniae | 40 | Specimens inoculated into BACTEC Peds Plus/F blood culture bottles and they were incubated at 35 degrees celcius in BACTECTM 9050  instrument within 2 hours of their collections.  Positive cultures were flagged and subcultured immediately onto sheep blood agar plates and incubated in a 5% CO2  incubator. S. pneumoniae isolates were identified on the basis of colony morphology, gram staining, susceptibility to optochin and bile solubility tests, which were done by standard methods.  Qualitative typing/groupin g was performed by doing the capsular reaction test (Neufeld test). | CLSI (susceptible, intermediate, or resistant) | Single centre study, moderate sample size, AST not disaggregated by specimen type, no strategy for duplicates | D | Prospective, single centre, small sample size, isolates further serotyped, AST not disaggregated by specimen type, no strategy for duplicates | C | Accreditation details not provided, EQA participation confirmed, organism ID and AST methodology completely described, AST not disaggregated by specimen type, isolates further serotyped, multidrug resistance pertaining to serotyped strains well described |
| --- | --- | --- | --- | --- | --- | --- | --- | --- | --- | --- | --- | --- | --- | --- | --- | --- | --- | --- | --- | --- |

| **Senanayak e** | 2014 | Outbreak of bloodstream infection with extended- spectrum beta- lactamase- producing Klebsiella pneumoniae at a teaching hospital. | Cohort | Prospective | Sri Lanka | Urban tertiary | 0 | 36 | 145 | HA | Blood | Klebsiella pneumoniae | 36 | Blood culture samples showing evidence of growth on overnight incubation at 37oC were promptly sub- cultured into MacConkey, Blood and chocolate agar media and incubated. The clinical isolates were identified by Gram’s staining, colony characteristics, and the findings were confirmed by the API 20E system. | CLSI | Small patient numbers, presumed prospective, single centre, brief report only, data not disaggregated by age/gender, AMR/MDR not well described, no strategy for duplicates described | D | Brief report, small patient numbers, presumed prospective, single centre, data not disaggregated by age/gender, AMR/MDR  not well described, no strategy for duplicates described | D | Accreditation details not provided, EQA participation not confirmed, organism ID and AST methodology incompletely described, AMR/MDR not well described |
| --- | --- | --- | --- | --- | --- | --- | --- | --- | --- | --- | --- | --- | --- | --- | --- | --- | --- | --- | --- | --- |

| **Shankar** | 2018 | Molecular characterisatio n for clonality and transmission dynamics of an outbreak of Klebsiella pneumoniae amongst neonates in a tertiary care centre in South India. | Cohort | Prospective | India | Urban tertiary | 0 | 13 | Nil | HA | Blood | Klebsiella pneumoniae | 13 | K.  pneumoniae isolates from blood culture were identified by standard biochemical methods | CLSI | Small patient numbers, cohort study, presumed prospective, single centre, data not disaggregated by age/gender | D | Small patient numbers, cohort study, presumed prospective, single centre, data not disaggregated by age/gender, strategy for duplicates well described, clinical syndromes/o utcomes well described | C | Accreditation details not provided, EQA participation not confirmed, organism ID and AST methodology well described, AMR/MDR well described, isolates further serotyped by: molecular characterisation, multilocus sequence typing, and Inc plasmid typing |
| --- | --- | --- | --- | --- | --- | --- | --- | --- | --- | --- | --- | --- | --- | --- | --- | --- | --- | --- | --- | --- |

| **Tagare** | 2010 | Multidrug resistant Klebsiella pneumoniae in NICU - what next? trend of antibiotic resistance. | Observatio nal cohort | Prospective | India | Urban tertiary | 0 | 115 | 1383 | HA | Blood | Klebsiella pneumoniae, non- Klebsiella gram- negative bacteria: E. coli, A. baumani, B. cepacia, P. aerogenosa, Enterobacter species, Enterococcus species, Aeromon. | 115 | The method used for detection of bacteria was BacT/Alert PF culture bottles 43-03053 with BacT/Alert Microbial Detection System. All blood cultures are observed for 72h, before they are reported as sterile.  Once growth is detected, the suspension of the organisms is plated to culture plates (blood agar and Mac- Conkey agar plates) and growth is assessed after 24h. The colony morphology is assessed and suspension prepared from the culture is submitted for automated biochemical assessment using VITEK 2 for organism identification. | Mini active pharmaceutical ingredient method (Biomerieux) | Presumed prospective, small sample size, nil strategy for duplicates, age/gender data not disaggregated, antibiotic resistance pattern of non-Klebsiella gram-negative bacteria not disaggregated per bacterium | D | Observational report, small sample size, presumed prospective, nil strategy for duplicates, age/gender data not disaggregated  , clinical outcomes discussed, data given per time frame thus temporal patterns observed | C | Accreditation details not provided, EQA participation not confirmed, organism ID and AST methodology well described, MDR quinolone and carbapenem resistant |
| --- | --- | --- | --- | --- | --- | --- | --- | --- | --- | --- | --- | --- | --- | --- | --- | --- | --- | --- | --- | --- |

| **Vala** | 2016 | Resistance Patterns of Typhoid Fever in Children: A Longitudinal Community- Based Study. | Longitudin al | Retrospective | India | Urban tertiary | 5mo - 18yo | 61 | Nil | CA | Blood | Salmonella typhi and S. paratyphi | 61 | Blood was collected in BacT/ALERT 3D culture bottles (bioMeuriex) and sent to the labaratory within 12 hours. Isolates were identified by standard biochemical methods. | CLSI | Small patient numbers, single centre, data not disaggregated by age/gender, no strategy for duplicates described | D | Longitudinal retrospective study, small sample size, nil strategy for duplicates, age/gender data not disaggregated  , clinical outcomes discussed | C | Accreditation details not provided, EQA participation not confirmed, organism ID and AST methodology completely described, no ID or AST errors detected |
| --- | --- | --- | --- | --- | --- | --- | --- | --- | --- | --- | --- | --- | --- | --- | --- | --- | --- | --- | --- | --- |

| **Verghese** | 2017 | Increasing incidence of penicillin- and cefotaxime- resistant Streptococcus pneumoniae causing meningitis in India: Time for revision of treatment guidelines? | Cohort | Prospective | India | Urban tertiary | Data extra cted for 0 - 16yo | 417  (data extract ed for 0 -  16yo) | Nil | CA | Blood, CSF | Streptococcu s pneumoniae | 417 | Pneumococcal isolation and confirmation were based on standard laboratory protocols. MIC testing of isolates was done by agar dilution from 2008 to 2010  and with VITEK System 2 (BioMerieux) from 2011 to  2016. | CLSI | Single centre, AST not disaggregated by age/gender, no strategy for duplicates, two different methods for testing susceptibility | D | Prospective, single centre, large sample size, isolates further serotyped, AST not disaggregated by age/gender, no strategy for duplicates, two different methods for testing susceptibility | C | Accreditation details not provided, EQA participation not confirmed, organism ID and AST methodology completely described, no ID or AST errors detected, isolates further serotyped, two different methods for testing susceptibility |
| --- | --- | --- | --- | --- | --- | --- | --- | --- | --- | --- | --- | --- | --- | --- | --- | --- | --- | --- | --- | --- |

| **Nazir** | 2019 | Multidrug- resistant Acinetobacter septicemia in neonates: A study from a teaching hospital of Northern India. | Cohort | Prospective | India | Urban tertiary | Neon ate | 49 | 357 | Not specifie d | Blood | *Acinetobacte r spp.* | 49 | Blood samples collected and cultured using BacTAlert3D. Samples were incubated for 7 days. Gram stain was carried out on positive bottles and inoculated onto blood agar and MacConkey agar plates for 24h at 37 deg. | CLSI | Focus on only one species. No differentiation between HA and CA. | D | Prospective, good sample size, appropriate denominator. No mention of HA vs CA. No mention of prior abx use. No mention how duplicates were managed.  Good discussion surrounding MDR.  Minimal clinical information | C | No accreditation/EQA not mentioned  Organism ID/AST methodology described. No ID or AST errors detected |
| --- | --- | --- | --- | --- | --- | --- | --- | --- | --- | --- | --- | --- | --- | --- | --- | --- | --- | --- | --- | --- |

| **Parajuli** | 2017 | Evaluating the trends of bloodstream infections among pediatric and adult patients at a teaching hospital of Kathmandu, Nepal: Role of drug resistant pathogens | Cross- Sectional | Prospective | Nepal | Urban tertiary | 0-14  years | 90  includi ng (2 citroba cter spp., and 2 entero bacter spp. No AMR) | 960 | Both | Blood | *S.aureus Enterococcus spp.*  *Salmonella enterica*  *E. Coli K.*  *pneumoniae*  *P. aeruginosa Acinteobacte r spp. Citrobacter spp Enterobacter spp.* | 86 | At the onset of fever (>37∘C) or in the presence of any clinical symptoms  compatible with infection, a blood culture specimen was taken with aseptic technique by cleansing of the collection site with 70% alcohol and subsequently followed by povidone iodine. One mL (for neonates), 5 mL (for children) of blood specimen were collected.  After incubation, at 37∘C for 24,  48, and 72  hours, blind subcultures were made on MacConkey agar and blood agar plates and observed for bacterial growth after 24hrs of aerobic  incubation at 37∘C. | CLSI | No mention of prior antibiotic use. | C | Repeated samples from the same patients were excluded.  Patients already on antibiotics were excluded. | B | Accreditation details not provided  EQA participation confirmed Organism ID and AST methodology completely described  No ID or AST errors detected |
| --- | --- | --- | --- | --- | --- | --- | --- | --- | --- | --- | --- | --- | --- | --- | --- | --- | --- | --- | --- | --- |

| **Patel** | 2014 | Blood culture isolates in neonatal sepsis and their sensitivity in Anand District of India | Cohort | Retrospective | India | Urban tertiary | Neon ate | 276  (positiv e from 226  newbo rns) includi ng 31 CoNS, 26  entero bacter spp., 15  pseudo monass pp., 5 entero occi spp., 27  candid a spp. and 14 others not specifie d | 1170 (note this includes duplicates as taken from 797 newborns) | Both | Blood | *Klebsiella pneumoniae CoNS*  *Enterobacter spp.*  *E. Coli Pseudomona s spp. Acinetobacte r spp. Enterococci spp. Staphylococc us aureus Candida spp. Other* | 214 | 1-2mL  collected for culture before starting antibiotics via venepuncture with aseptic precautions. Cultures done using BacT/ALERT PF  system. Cultures isolated for 16-24 hours. | Not defined | No AMR definition. No mention of duplication or prior antibiotic use. | C | Retrospective  .  Differentiatio n between early and late onset sepsis however no AMR  definition. No mention of duplication or prior antibiotic use. | E | No accreditation/EQA not mentioned  Organism ID/AST methodology not described. |
| --- | --- | --- | --- | --- | --- | --- | --- | --- | --- | --- | --- | --- | --- | --- | --- | --- | --- | --- | --- | --- |

| **Pokhrel** | 2018 | Bacteriological profile and antibiotic susceptibility of neonatal sepsis in neonatal intensive care unit of a tertiary hospital in Nepal | Cross- sectional | Retrospective | Nepal | Urban tertiary | Neon ates | 69  includi ng 23 klebsiel la spp., 14 CoNS, 13  entero bacter spp., 3 serratia rubida ea, 2 pseudo monass pp., 1 bacillus spp., 1 non- hemoly tic strep | 332 | Not specifie d | Blood | *Klebsiella spp.*  *Enterobacter spp.*  *Acinetobacte r spp.*  *Serratia rubidaea*  *E. Coli Pseudomona s spp. Bacillus spp. CoNS*  *S. Aureus Non- hemolytic strep* | 50 | Blood cultures were taken following disinfection of the skin. 1-3 mL were taken aseptically from a peripheral vein. Samples were incubated at 35 +/- 2 deg  for 5 days. | CLSI | Single centre which may reflect local demographics.  Small study population and limited yield of some pathogens. No mention of pre- antibiotic use. No mention of CA vs HA. | C | Retrospective  .  Differentiatio n between early and late onset sepsis. No mention of duplication or prior antibiotic use. | D | Accreditation details not provided  EQA participation not confirmed Organism ID and AST methodology completely described  No ID or AST errors detected |
| --- | --- | --- | --- | --- | --- | --- | --- | --- | --- | --- | --- | --- | --- | --- | --- | --- | --- | --- | --- | --- |

| **Rafi** | 2020 | Risk factors and etiology of neonatal sepsis after hospital delivery: A  case-control study in a tertiary care hospital of Rajshahi, Bangladesh. | Case control | Prospective | Banglad esh | Urban tertiary | Neoa ntes | 91  includi ng 8 staph saprop hyticus  , 3  staph epider mis and 1 strep viridan s | 284 | HA | Blood | *E. Coli Klebsiella pneumoniae*  *S. Aureus Staph saprophyticu s*  *Staph epidermis Strep viridans* | 87 | 0.5-1mL blood collected from clinically suspected neonates.  Blood cultures were incubated in BACT/Alert machine for 5 days. Positive samples were inoculated onto MacConkey agar plates and incubated at 35deg. | CLSI | Single centre study. Small sample size. Only HA, no menton of CA organisms.  Percentages not whole numbers used. Unclear if prospective or retrospective. | C | Differentiatio n between early and late onset sepsis. Small sample size. No mention duplication or prior antibiotic use. | C | Accreditation details not provided  EQA participation not confirmed Organism ID and AST methodology completely described  No ID or AST errors detected |
| --- | --- | --- | --- | --- | --- | --- | --- | --- | --- | --- | --- | --- | --- | --- | --- | --- | --- | --- | --- | --- |

| **Rose** | 2014 | Antimicrobial susceptibility profile of isolates from pediatric blood stream infections | Cohort | Prospective | India | Urban tertiary | 30  days - 15  years | 2015  (522  non- fermen ting gram negativ e bacilli other than pseudo monas excl; 128  pseudo monas spp.  200,  klebsiel la spp., 58  entero bacter spp. and 111  entero coccus spp incl). | 41457 | CA | Blood | Non- fermenting gram negative bacilli other than pseudomona s  Salmonella spp.  E. Coli Klebsiella spp. Pseudomona s spp. Enterobacter spp.  S. Aureus Enterococcus spp.  S.  Pneumoniae | 1493 | Blood cultures were inoculated and processed by BacT/ALERT followed by gram staining and sub- cultures on MacConkey agar and 5% sheep blood agar.  Biochemical identification of the pathogen was done using standard procedurs. | CLSI | Single centre study. No clinical inclusion/exclusion criteria. No mention prior abx use. No mention duplications. AST method not defined. | D | Single centre study. No clinical inclusion/excl usion criteria. No mention prior abx use. No mention duplications. AST method not defined. | D | Accreditation details not provided  EQA participation not confirmed Organism ID and AST methodology partially described No ID or AST errors detected |
| --- | --- | --- | --- | --- | --- | --- | --- | --- | --- | --- | --- | --- | --- | --- | --- | --- | --- | --- | --- | --- |

| **Roy** | 2017 | Pattern of Pediatric Bacterial Infection and Antibiotic Resistance in New Delhi. | Cohort | Retrospective | India | Urban tertiary | Childr en under 12  years | 1025  excl 254 CoNS,  incl 73 entero coccuss  pp. 39 klebsiel laspp., 36  entero bacter spp. | Not given | CA | Blood | *S. Aureus CoNS*  *Enterococcus spp.*  *E. Coli Acinetobacte r spp. Klebsiella spp. Enterobacter spp.* | 771 | Blood cultures were taken from children up to 12 years admitted with acute infections, sepsis or pneumonia. Blood cultures were incubated at 37 DEG FOR 7  days. | CLSI | No denominator. Single centre. No mention of how samples were collected or clinical data. Nomention of previous antibiotic use. No mention of CA vs HA. | D | No denominator. Single centre. No mention of how samples were collected or clinical data. Nomention of previous antibiotic use. No mention of CA vs HA. | C | Accreditation details not provided  EQA participation not confirmed Organism ID and AST methodology completely described  No ID or AST errors detected |
| --- | --- | --- | --- | --- | --- | --- | --- | --- | --- | --- | --- | --- | --- | --- | --- | --- | --- | --- | --- | --- |

| **Roy** | 2015 | Insight into neonatal septicaemic Escherichia coli from India with respect to phylogroups, serotypes, virulence, extended- spectrum-  $\beta$- lactamases and association of ST131 clonal group. | Cohort | Not specified | India | Urban tertiary | Neon ates | 67 | Not given | Not specifie d | Blood | *E. Coli* | 67 | Blood cultures were collected prior to antibiotic therapy when the neonates were suspected for having sesis. | CLSI | No denominator. No clear differentiation between hospital vs. community acquired. Small sample size considering duration of data collection. | C | No denominator. Multi centre data collection. No mention relating to previous antibiotic use. No mention of CA vs HA. | C | Accreditation details not provided  EQA participation not confirmed Organism ID and AST methodology completely described  No ID or AST errors detected |
| --- | --- | --- | --- | --- | --- | --- | --- | --- | --- | --- | --- | --- | --- | --- | --- | --- | --- | --- | --- | --- |

| **Roy** | 2013 | Neonatal septicaemia caused by diverse clones of Klebsiella pneumoniae & Escherichia coli harbouring blaCTX-M-15 | Cohort | Not specified | India | Urban tertiary | Neon ates | 26 | 405 with clinical  sepsis; 177 with culture postive sepsis | Both | Blood | *Klebsiella pneumoniae*  *E. Coli* | 26 | 1mL blood for culture was drawn with aseptic precautions from a peripheral vein of neonates with suspected sepsis.  Cultures were processed in BacT/ALERT 3D system (bioMe ́rieux, Marcy l’Etoile, France). For any culture which flagged positive, Gram stain was performed and subculture was done on appropriate medium based on the Gram stain: MacConkey agar and 5 per cent sheep blood agar for Gram-negative and Gram- positive organisms, respectively. Bottles were incubated in the system for up to seven days, at the end of which all negative bottles were subcultured once on blood agar before discarding. | CLSI | Small sample size. No clear discussion surrounding other organisms cultured. Small duration of data collection not allowing for data to reflect seasonal changes. | D | Single centre. Small duration of data collection. No mention of previous antibiotic use or duplications. | C | Accreditation details not provided  EQA participation not confirmed Organism ID and AST methodology completely described  No ID or AST errors detected |
| --- | --- | --- | --- | --- | --- | --- | --- | --- | --- | --- | --- | --- | --- | --- | --- | --- | --- | --- | --- | --- |

| **Sekar** | 2017 | Carbapenem- resistant Enterobacteria ceae in pediatric bloodstream infections in rural Southern India. | Cross- sectional | Retrospective | India | Urban tertiary | 0-12  years | 880  excl 333 CoNS  and 29 candid a, incl 67  entero coccuss pp., 4 Klebsiel la oxytoc a, 10  proteu s mirabili s, 4 proteu s vulgari s, 9 entero bacters pp., 18 citroba cter spp., 2 morga nellasp p., 3  provide ncia spp., 5 hafnia spp., 2 serratia spp., 121  non- fermen ting gram- negativ e bacilli, 12  gram- negativ e cocci/c occoba cilli, 29 candid a spp. | 1932 | Not specifie d | Blood | *Staph aureus CoNS*  *Streptococcu s spp.*  *Enterococcus spp.*  *E. Coli Klebsiella pneumoniae Klebsiella oxytoca Proteus mirabilis Proteus vulgaris Enterobacter spp. Citrobacter spp. Morganella spp. Providencia spp.*  *Hafnia spp. Serratia spp. Salmonella spp.*  *Shigella spp. Pseudomona s aeruginosa Candida spp.* | 518 | Blood cultures taken from children age 0- 12years were included in the study. Blood culture and the identification of isolate was performed as per the standard procedure | CLSI | Single centre study in tertaiary NICU not necessarily reflective of general population. Most patients were referred for blood culture only after the failure of empiric therapy, which might have prejudiced the high resistance rate observed in this study. No mention duplications. No clear distinguishing between CA or HA. | D | Single centre study in tertaiary NICU not necessarily reflective of general population. Most patients were referred for blood culture only after the failure of empiric therapy, which might have prejudiced the high resistance rate observed in this study. No mention duplications. No clear distinguishing between CA or HA. | C | Accreditation details not provided  EQA participation not confirmed Organism ID and AST methodology completely described  No ID or AST errors detected |
| --- | --- | --- | --- | --- | --- | --- | --- | --- | --- | --- | --- | --- | --- | --- | --- | --- | --- | --- | --- | --- |

| **Shahunja** | 2020 | Clinical and laboratory characteristics of children under five hospitalized with diarrhea and bacteremia. | Cross- sectional | Retrospective | Banglad esh | Urban tertiary. | 0-5  years | 401  includi ng 35 pseudo monass pp., 22 strepto coccuss pp., 9 campyl obacte rspp., 65  staphyl ococcu sspp., (27  others excl) | 4141 | CA | Blood | *Salmonella typhi Staphylococc us spp.*  *Pseudomona s spp.*  *E. Coli Klebsiella spp. Acinetobacte r spp. Streptococcu s spp. Salmonella paratyphi Campylobact er spp.*  *Non- typhoidal salmonella Enterococcus spp.*  *Shigella spp. Others* | 374 | Patients admitted wth diarrhoea who based on clinical judgements were selected for blood culture. 2mL of venous blood was collected via aseptic technique and seeded directly into BacT/ALERT culture bottles and entered into the BacTAlert 3D system | CLSI | Resource limited single centre study. Blood cultures were collected based on clinical judgment which allows for bias.  Hence actual rates of bacteraemia are not accurate.Details surrounding prior antibiotic use was not available. | D | Single centre study. Unable to correlate findings with clincial history. No mention prior antibiotuc use or duplication | C | Accreditation details not provided  EQA participation not confirmed Organism ID and AST methodology completely described  No ID or AST errors detected |
| --- | --- | --- | --- | --- | --- | --- | --- | --- | --- | --- | --- | --- | --- | --- | --- | --- | --- | --- | --- | --- |

| **Sharma** | 2013 | Staphylococcus aureus- the predominant pathogen in the neonatal ICU of a tertiary care hospital in Amritsar, India | Cohort | Retrospective | India | Urban tertiary | Neon ates | 131  includi ng 30 CoNS  (excl), 4  entero bacter cloacae  , 2  citroba cter and 1 candid a (excl) | 311 | Not specifie d | Blood | *S. Aureus CoNS*  *Klebsiella pneuoniae Acinetobacte r baumannii*  *E. Coli Enterobacter cloacae Citrobacter diversus Psedomonas aeruginosa Candida* | 100 | All blood cultures were taken from peripheral veins following aseptic preparation prior to any antibiotic therapy commencing. 2-3 mL of blood was inoculated into brain- heart infusion broth and then incubated at 37 deg.  Subcultures were done on blood and MacConkey’s agar plates on days 1,2,3,5,7  and 10. | CLSI | Single centre study in tertaiary NICU not necessarily reflective of general population. No mention duplications. No clear distinguishing between CA or HA. | C | Single centre study in tertaiary NICU not necessarily reflective of general population. No mention duplications. No clear distinguishing between CA or HA. MDR  not mentioned. | C | Accreditation details not provided  EQA participation not confirmed Organism ID and AST methodology completely described  No ID or AST errors detected |
| --- | --- | --- | --- | --- | --- | --- | --- | --- | --- | --- | --- | --- | --- | --- | --- | --- | --- | --- | --- | --- |

| **Shrestha** | 2015 | Bacterial meningitis in children under 15 years of age in Nepal | Cohort | Prospective | Nepal | Urban tertiary | 0-15  years | 18  includi ng 3 group Bstrept ococcu s, 1 pseudo monass pp. | 252 | Not specifie d | CSF | *H. Influenzae Group B Streptococcu s*  *E. Coli Pseudomona s spp.*  *S.*  *Pneumoniae N.*  *Meningitidis* | 18 | After obtaining the consent, the CSF specimens were collected by medical officers in a sterile tube. The vol- ume and gross appearance i.e., consistency, presence of blood and the color of CSF were noted. CSF specimens were transportedto the laboratory within half an hour. CSF specimen was inoculated in MacConkey agar (MA), blood agar (BA) and chocolate agar (CA) plates.  Then incubated overnight at 37 deg. | CLSI | Single centre study. Small sample size. No mention duplications or prior antibiotic use (relevant for those having LPs performed). No clinical data provided. | D | Single centre study. Focus only on CSF with overall small numerator sample size. No mention duplications or prior antibiotic use (relevant for those having LPs performed). No clinical data provided. | C | Accreditation details not provided  EQA participation not confirmed Organism ID and AST methodology completely described  No ID or AST errors detected |
| --- | --- | --- | --- | --- | --- | --- | --- | --- | --- | --- | --- | --- | --- | --- | --- | --- | --- | --- | --- | --- |

| **Thapa** | 2019 | Changing trend of neonatal septicemia and antibiotic susceptibility pattern of isolates in Nepal. | Cohor | Prospective | Nepal | Urban tertiary | Neon ates | 56  includi ng 6 CoNS  and 5 entero bacters pp., 2 citroba cter spp., 3 pseudo monass pp. | 516 | Both | Blood | *Acinetobacte r spp*  *S. Aureus CoNS*  *E. Coli Enterobacter spp. Klebsiella pneumoniae Pseudomona s spp. Citrobacter spp. Salmonela paratyphi A* | 50 | Neoates with clinical manifestation of septicaemia had 1-2 ml of blood was drawn aseptically before starting antimicrobial therapy and directly inoculated into Brain Heart Infusion broth (BHI) (HiMedia, India) in a ratio of blood:BHI of 1:5. The blood culture bottles were immediately sent to the microbiology laboratory and incubated at 37∘C for 24  hrs and  subcultured on MacConkey agar, blood agar, and chocolate agar (HiMedia, India) daily for 7 days. | CLSI | Antimicrobial susceptibility not provided for all organisms. Single centre study in  non-tertiary centre. No mention duplications. | D | Antimicrobial susceptibility not provided for all organisms. Single centre study in non- tertiary centre. No mention duplications. | C | Accreditation details not provided  EQA participation not confirmed Organism ID and AST methodology completely described  No ID or AST errors detected Percentages not whole numbers provided. |
| --- | --- | --- | --- | --- | --- | --- | --- | --- | --- | --- | --- | --- | --- | --- | --- | --- | --- | --- | --- | --- |

| **Tiwari** | 2013 | A study on the bacteriological profile and antibiogram of bacteremia in children below 10 years in a tertiary care hospital in bangalore, India. | Cohort although not clear | Not specified | India | Urban tertiary | 1 day  to 10 years | 32  includi ng 14 Klebsiel la spp., 3 CoNS  (excl), 1  citroba cter freundi i | 128 | Not specifie d | Blood | *Klebsiella spp.*  *Staphylococc us aureus CoNS*  *Pseudomona s aeruginosa Salmonella typhi*  *E. Coli Acinetobacte r baumanii Citrobacter freundii* | 29 | 1mL for neonates and 5mL for children were collected and inoculated. Blood cultures were inoculated into 10 and 50 ml, respectively, of brain heart infusion broth (1:10 dilution). The culture bottles were incubated at 37oC  aerobically and periodic subcultures were done onto Mac Conkey’s agar, blood agar and chocolate agar after overnight incubation on day 3, day 4 and finally on day 7 | CLSI | Single centre study. Small sample size. No mention duplications or prior antibiotic use. No clinical data provided. | D | Single centre study. Small sample size. No mention duplications or prior antibiotic use. No clinical data provided. | C | Accreditation details not provided  EQA participation not confirmed Organism ID and AST methodology completely described  No ID or AST errors detected |
| --- | --- | --- | --- | --- | --- | --- | --- | --- | --- | --- | --- | --- | --- | --- | --- | --- | --- | --- | --- | --- |

| **Yadav** | 2018 | Bacteriological profile of neonatal sepsis and antibiotic susceptibility pattern of isolates admitted at Kanti Children's Hospital, Kathmandu, Nepal | Cross- sectional | Prospective | Nepal | Urban tertiary | <1  mont h | 59  includi ng 6 CoNS  (excl) and 6 entero bacters pp., 1 citroba cter spp. | 350 | Not specifie d | Blood | *S.aureus K.*  *pneumoniae. E.coli Enterobacter spp.*  *Acinetobacte r spp*  *P. aeruginosa Citrobacter spp*  *CoNS*  *S. typhi* | 53 | Blood samples (1–2 ml) were collected from suspected neonates  and inoculated directly into brain heart infusion (BHI; HiMedia, M210) broth and incubated at  37 °C for 5–7 days. After incubation, subcultured from BHI broth was performed  on blood agar and MacConkey agar. The MA plates were incubated aerobically and BA plates were incubated anaerobically. The pure isolates obtained from subcultured plates were identifed by following standard microbiologica l  techniques which include studies of colony morphology, Gram-staining reactions and various biochemical properties. | CLSI | Short study recruitment period that may not have encompassed seasonal change. No definition of outborne vs inborne infants.  Limited population group to those admitted to Kanti Children's Hospital. No break-down of organisms by age range. | C | Prospective study, but small sample size, short recruitment period.  Differentiatio n made between early and late onset sepsis. No clear discussion surrounding histry of antibiotic use. | C | No accreditation/EQA not mentioned  Organism ID/AST methodology described. No ID or AST errors detected |
| --- | --- | --- | --- | --- | --- | --- | --- | --- | --- | --- | --- | --- | --- | --- | --- | --- | --- | --- | --- | --- |

| **Azizah Abu, N** | 2016 | Community- acquired Bacteremia in paediatrics: Epidemiology, aetiology and patterns of antimicrobial resistance in a tertiary care centre, Malaysia | Case series | Retrospective | Malaysi a | Urban tertiary | 0-13 | 222 | 2134 | CA | Blood | *Acinetobacte r* spp.  *Escherichia coli Streptococcu s agalactiae Haemophilus influenzae Klebsiella* spp.  *Neisseria meningitidis Salmonella* spp.  *Pseudomona s aeruginosa Staphylococc us aureus Streptococcu s pneumoniae Streptococcu s pyogenes* | 173 | Organism identification via API and VITEK. | CLSI | Minimal breakdown of each clinical syndrome for each pathogen and no breakdown of each sensitivity for each pathogen. | C | Single site, retrospective, observational study. Risk of bias. | C | No accreditation/EQA details, MDR not discussed or defined. Organism ID and AST methods described. No clear errors. |
| --- | --- | --- | --- | --- | --- | --- | --- | --- | --- | --- | --- | --- | --- | --- | --- | --- | --- | --- | --- | --- |

| **Anderson, M** | 2014 | Epidemiology of bacteremia in young hospitalized infants in Vientiane, Laos, 2000-2011. | Case series | Prospective | Laos | Urban tertiary | 0-0.5 | 85 | 1438 | CA | Blood | *Acinetobacte r* spp.  *Escherichia coli Streptococcu s agalactiae Klebsiella* spp.  *Pseudomona s aeruginosa Staphylococc us aureus Streptococcu s pneumoniae Streptococcu s pyogenes Salmonella* spp. | 72 | Not defined | CLSI | No reporting of organism identification method. No specific reporting of AST method except for ESBL | C | Single site, prospective, observational study. Risk of bias. | D | Accreditation details not provided EQA participation not confirmed Organism ID and AST methodology only partially described. No ID or AST errors detected |
| --- | --- | --- | --- | --- | --- | --- | --- | --- | --- | --- | --- | --- | --- | --- | --- | --- | --- | --- | --- | --- |

| **Arrifin, N** | 2012 | Comparison of antimicrobial resistance in neonatal and adult intensive care units in a tertiary teaching hospital. | Case series | Retrospective | Malaysi a | Urban tertiary | 0 | 742 | 742 | HA | Blood | *Acinetobacte r* spp.  *Escherichia coli Klebsiella* spp.  *Pseudomona s aeruginosa Staphylococc us aureus* | 252 | Processing via CLSI  guidelines. Identification based on a conventional system and/or an API system. | CLSI | Retrospective case series. No differentiation of CoNS whether clinical sepsis or not. | C | Single site, retrospective, case series study. Risk of bias. | B | Accreditation details provided. EQA participation not confirmed Organism ID and AST methodology described. No ID or AST errors detected |
| --- | --- | --- | --- | --- | --- | --- | --- | --- | --- | --- | --- | --- | --- | --- | --- | --- | --- | --- | --- | --- |
| **Bao, Y** | 2019 | The changing phenotypes and genotypes of invasive pneumococcal isolates from children in Shenzhen during 2013-  2017. | Case series | Retrospective | China | Urban tertiary | 0-13 | 94 | 94 | N/A | Blood CSF  joint aspirate | *Streptococcu s pneumoniae* | 94 | Optochin sensitivity test, bile solubility test, Omni serum assay. | CLSI | Sampling method not described - implies recruited all invasive pneumococcal isolates. | C | Single site. Retrospective  . Risk of bias. | C | Accreditation details not provided. EQA participation not confirmed. Organism ID and AST methodology completely described. No ID or AST errors detected |

| **Wang, C** | 2020 | Analysis of Pathogen Distribution and Its Antimicrobial Resistance in Bloodstream Infections in Hospitalized Children in East China, 2015-  2018 | Case series | Retrospective | China | Urban tertiary | 0-13 | 14107 | N/A | HA | Blood | *Acinetobacte r* spp.  *Escherichia coli Streptococcu s agalactiae Haemophilus influenzae Klebsiella* spp.  *Staphylococc us aureus Streptococcu s pneumoniae Streptococcu s pyogenes Salmonella* spp. | 4633 | VITEK-2,  Microscan WalkAway-96 Plus, Phoenix- 100 System or mass spectrometry | CLSI | Error in Staph numbers | C | Multi-site, retrospective study with large numbers. X1 Imprecision noted (S. aureus difference between tables). Risk of bias. | C | Accreditation details not provided. EQA participation not confirmed. Organism ID and AST fully described. No ID or AST errors detected |
| --- | --- | --- | --- | --- | --- | --- | --- | --- | --- | --- | --- | --- | --- | --- | --- | --- | --- | --- | --- | --- |

| **Zhao, C** | 2020 | Investigation of antibiotic resistance, serotype distribution, and genetic characteristics of 164 invasive streptococcus pneumoniae from North China between April 2016 and  October 2017 | Case series | Prospective | China | Urban tertiary | 0-17 | 164 | N/A | N/A | Blood CSF  pleural fluid | *Streptococcu s pneumoniae* | 164 | Culture and biochemistry/s elective agars (optochin, bile solubility, alpha haemolysis) | CLSI | No assessment of MDR. | C | Prospective, multi-center study with relatively small numbers. Calculation errors (AST reported in %  - meningitis calculations not clear).. Risk of bias. | C | Accreditation details not provided. EQA participation not confirmed (but use quality control specimen). Organism ID and AST fully described. No ID or AST errors detected. |
| --- | --- | --- | --- | --- | --- | --- | --- | --- | --- | --- | --- | --- | --- | --- | --- | --- | --- | --- | --- | --- |

| **Chheng, K** | 2013 | A prospective study of the causes of febrile illness requiring hospitalization in children in Cambodia. | Case series | Prospective | Cambod ia | Urban non- tertiary | 0-15 | 85  (75 BC,  CSF 10) | 1225  (1212 BC) | CA | Blood CSF | *Acinetobacte r* spp.  *Escherichia coli Klebsiella* spp.  *Haemophilus influenzae Neisseria meningitidis Pseudomona s aeruginosa Staphylococc us aureus Streptococcu s pneumoniae Streptococcu s pyogenes Salmonella* spp. | 79 | Agar then API test kits.  NAATs for S. pneumoniae, Hib, N. meningitidis and S. suis | CLSI | Large prospective study but very minimal AMR data given for isolates. | C | Single site, prospective, observational high quality study. Risk of bias. Minimal details of AMR data despite testing i.e publication bias  ?sufficient to drop to D | C | Accreditation details not provided. EQA participation not confirmed. Organism ID and AST fully described. No ID or AST errors detected |
| --- | --- | --- | --- | --- | --- | --- | --- | --- | --- | --- | --- | --- | --- | --- | --- | --- | --- | --- | --- | --- |

| **Li, C** | 2018 | Clinical characteristics and etiology of bacterial meningitis in Chinese children >28 days of age, January 2014- December 2016: a  multicenter retrospective study. | Case series | Retrospective | China | Urban tertiary and urban- non tertiary | 0.5-  18 | 374 (BC 163, CSF 211) | 837 | N/A | Blood CSF | *Escherichia coli Streptococcu s agalactiae Haemophilus influenzae Neisseria meningitidis Staphylococc us aureus Streptococcu s pneumoniae Salmonella* spp. | 217 | Not stated in detail. Only mentions, culture, gram stain and PCR in passing. | CLSI | No mention of Organism ID, AST method, CA/HA | C | Retrospective  , multi-centre observational study. Risk of bias. Minimal details on Organism ID/AST  methodology. | D | Accreditation details not provided. EQA participation not confirmed. Organism ID and AST not described in detail. No ID or AST errors detected |
| --- | --- | --- | --- | --- | --- | --- | --- | --- | --- | --- | --- | --- | --- | --- | --- | --- | --- | --- | --- | --- |

| **Dong, Y** | 2017 | Group B Streptococcus causes severe sepsis in term neonates: 8 years experience of a major Chinese neonatal unit. | Case series | Retrospective | China | Urban tertiary | 0 | 43 | N/A | N/A | Blood CSF | *Streptococcu s agalactiae* | 43 | BacT/ALERT System for blood. CSF: blood agar, gram stain, catalase, CAMPT and lancefield grouping | Not stated | Small numbers. No mention of AMR definition | C | Retrospective  , single centre observational study. Risk of bias.  Information of ID/AST but reference used not mentioned | D | Accreditation details not provided. EQA participation not confirmed. Organism ID and AST fully described but no information on which reference. No ID or AST errors detected |
| --- | --- | --- | --- | --- | --- | --- | --- | --- | --- | --- | --- | --- | --- | --- | --- | --- | --- | --- | --- | --- |

| **Dong, F** | 2018 | Epidemiology of carbapenem- resistant Klebsiella pneumoniae bloodstream infections in a Chinese Children's Hospital: predominance of New Delhi metallo-beta- lactamase-1. | Case series | Retrospective | China | Urban tertiary | Paedi atric Patie nts (not specif ied) | 164 | N/A | N/A | Blood | *Klebsiella*  spp. | 164 | VITEK-2 &  Phoenix-100 | CLSI, EUCAST for  Colistin | Not MDR definition. Only extended AST on Carapenem resistant organisms. Did not state numbers of Carapenem resistant organisms tests for exam Abx only percentages | C | Retrospective  , small single center, observational study. Risk of bias. | C | Accreditation details not provided. EQA participation not confirmed. Organism ID and AST fully. No ID or AST errors detected |
| --- | --- | --- | --- | --- | --- | --- | --- | --- | --- | --- | --- | --- | --- | --- | --- | --- | --- | --- | --- | --- |

| **Fox-Lewis, A** | 2018 | Antimicrobial Resistance in Invasive Bacterial Infections in Hospitalized Children, Cambodia, 2007-2016. | Case series | Retrospective | Cambod ia | Urban non- tertiary | Paedi atric Patie nts (not specif ied) | 1341 | 39050 | Both | Blood CSF | *Escherichia coli Klebsiella* spp.  *Acinetobacte r* spp.  *Staphylococc us aureus Streptococcu s pneumoniae Salmonella* spp.  *Pseudomona s aeruginosa Streptococcu s pyogenes Haemophilus influenzae Neisseria meningitidis* | 1233 | Referenced papers from same site: API and routine methods. | CLSI | No MDR definition | C | Retrospective  ,large single center, observational study. Risk of bias. | C | Accreditation details not provided. EQA participation not confirmed. Organism ID and AST fully. No ID or AST errors detected |
| --- | --- | --- | --- | --- | --- | --- | --- | --- | --- | --- | --- | --- | --- | --- | --- | --- | --- | --- | --- | --- |

| **Shen, H** | 2019 | The etiology of acute meningitis and encephalitis syndromes in a sentinel pediatric hospital, Shenzhen, China | Case series | Prospective | China | Urban tertiary | Paedi atric Patie nts (not specif ied) | 70 | 171 | N/A | Blood CSF | *Streptococcu s agalactiae Streptococcu s pneumoniae Escherichia coli Haemophilus influenzae Neisseria meningitidis Pseudomona s aeruginosa Klebsiella* spp.  *Acinetobacte r* spp.  *Staphylococc us aureus* | 56 | VITEK-2,  Serotyping, PCR, Lateral flow assay, NGS | CLSI | Calculation errors | D | Prospective, small single center, observational study. Risk of bias. Errors in calculations. | C | Accreditation details not provided. EQA participation not confirmed. Organism ID and AST fully described. No ID or AST errors detected |
| --- | --- | --- | --- | --- | --- | --- | --- | --- | --- | --- | --- | --- | --- | --- | --- | --- | --- | --- | --- | --- |

| **Jiang, H** | 2017 | Prevalence and antibiotic resistance profiles of cerebrospinal fluid pathogens in children with acute bacterial meningitis in Yunnan province, China, 2012-  2015. | Case series | Prospective | China | Urban tertiary | 0-12 | 179 | N/A | N/A | CSF | *Streptococcu s pneumoniae Streptococcu s agalactiae Staphylococc us aureus Escherichia coli Haemophilus influenzae Salmonella* spp.  *Klebsiella*  spp. *Pseudomona s aeruginosa Acinetobacte r* spp. | 135 | Immediately after collection, each CSF specimen was centrifuged at 1500 rpm for  15 minutes. The supernatant was removed, and the sediment was analyzed by Gram a stains if the  patient's white blood cell count was  >10/mm3. In addition, cerebrospinal fluid (CSF) specimens were inoculated into PEDS Plus bottles and cultivated using the BD BACTEC™ FX  system (BD  Diagnostics, Sparks, MD). Gram stain results were used to guide subcultures and susceptibility testing, which was performed using the disc diffusion method.  Specimens were subcultured onto 5% sheep blood agar and chocolate agar plates. All culture plates were incubated  at 37 degree C for 24±48 hours in a 5% carbon dioxide environment and at room temperature for bacterial organisms, respectively. Bacterial isolates were identified using the Vitek-32  system (BioMerieux). | CLSI | No details of how cases were identified or excluded  MDR not defined | D | Single site observational study Inclusion process not documented, not clear if consecutive cases.  Significant risk of bias. | E | Overt ID and/or AST errors detected/strongly suspected |
| --- | --- | --- | --- | --- | --- | --- | --- | --- | --- | --- | --- | --- | --- | --- | --- | --- | --- | --- | --- | --- |

| **Cai, K** | 2018 | Clinical characteristics and antimicrobial resistance of pneumococcal isolates of pediatric invasive pneumococcal disease in China | Case series | Prospective | China | Urban tertiary | 0-14 | 123 | 20719 | N/A | Blood CSF  synovial fluid pericardial fluid  pleural fluid peritoneal fluid | *Streptococcu s pneumoniae* | 123 | Samples were inoculated on blood-agar plates and incubated at 35°C, 5% CO2  incubators for 24 hours. *S. pneumoniae* isolates were identified by colony morphology on blood agar and optochin test, and confirmed by matrix- assisted laser desorption/ion ization time- of-flight (MALDI-TOF)  mass spectrometry (Microflex LT; Bruker, Billerica, MA, USA). For MALDI-TOF  analysis, bacterial proteins from blood cultures were extracted using a MALDI Sepsityper kit (Bruker). Each purified blood culture extract (1 μL) was transferred to an individual spot on the Bruker 96-spot target plate and covered with a 1 μL α- cyano-4- hydroxycinna mic acid matrix (Bruker). The target plate was then read and analyzed by the Bruker Microflex LT system. A protein profile of each specimen with m/z values of 3,000–15,000  was generated based on a minimum of 240 laser-shot measurements  . Profiles were further analysed using Biotyper 3.0 software (Bruker) in blood-culture mode according to the manufacturer’ s recommendati  on. | CLSI | Sampling strategy not well defined. Number of excluded cases after consent sought not reported. | D | Observational study.  Sampling strategy not clear. Small sample size. Significant risk of bias. | D | Accreditation details not provided, organism ID/AST methodology partially described, MDR not defined. No overt ID or AST errors detected. |
| --- | --- | --- | --- | --- | --- | --- | --- | --- | --- | --- | --- | --- | --- | --- | --- | --- | --- | --- | --- | --- |

| **Li, J** | 2019 | Molecular characteristics of group B Streptococcus isolates from infants in southern mainland China. | Case series | Retrospective | China | Urban tertiary | 0-0.5 | 93 | N/A | N/A | Bloods CSF  Joint fluid | *Streptococcu s agalactiae* | 93 | VITEK 2 COMPACT  (BioMerieux, Marcy L’Etoile, France).  Isolates centralized in laboratory of Guangzhou Women and Children’s Medical Center for analysis, where they were cultured at 37 °C in 5% CO2 in trypticase soy agar supplemented with 5% sheep blood. ATCC 2592 and  ATCC 49619  were used as quality control bacteria. | CLSI | Sampling strategy not well defined. Number of excluded cases not included.  Denominator not included. Not differentiate between CA/HA. Duplicates not reported. No MDR definition. | D | Observational study.  Sampling strategy not clear. Small sample size. Significant risk of bias. | C | Accreditation details not provided. EQA participation not confirmed. Organism ID and AST methodology completely described. No ID or AST errors detected |
| --- | --- | --- | --- | --- | --- | --- | --- | --- | --- | --- | --- | --- | --- | --- | --- | --- | --- | --- | --- | --- |

| **Kang, L** | 2016 | Molecular epidemiology of pneumococcal isolates from children in China. | Case series | Retrospective | China | Urban tertiary | 0-11 | 51 | N/A | N/A | Blood CSF  Bone marrow Pleural fluid pleural fluid (x2 "pus") | *Streptococcu s pneumoniae* | 51 (note x2 from pus) | Not stated | CLSI | Sampling strategy not defined.  Number of excluded cases not included.  Denominator not included. Not differentiate between CA/HA. Duplicates not reported. MDR not defined. Specimen processing / target Iorg ID process for IPD not included. | D | Observational study.  Sampling strategy not defined.  Antimicrobial methods poorly described.  Small sample size.  Significant risk of bias. | E | Overt ID and/or AST errors detected/strongly suspected |
| --- | --- | --- | --- | --- | --- | --- | --- | --- | --- | --- | --- | --- | --- | --- | --- | --- | --- | --- | --- | --- |

| **Guo, L** | 2016 | Clinical and pathogenic analysis of 507 children with bacterial meningitis in Beijing, 2010-  2014. | Case series | Retrospective | China | Urban tertiary | 0-5-  15 | 220  pathog en positiv e | 507 | N/A | CSF  Blood | *Streptococcu s pneumoniae Pseudomona s aeruginosa Haemophilus influenzae Neisseria meningitidis Salmonella* spp*.*  *Staphylococc us aureus Escherichia coli Streptococcu s agalactiae* | 147 | CSF samples were obtained aseptically from each participant through lumbar puncture. Up to 1 ml of CSF was collected into a sterile tube. Samples were sent immediately to the hospital laboratory for a cell count, Gram staining, and bacterial culture, as well as to measure glucose and protein levels. Twenty-four hours after incubation at 35 +/- 2  degrees C in 5% CO2,  bacterial isolates were identified by colony morphology analysis and growth requirements. After identification, isolates of S. pneumoniae were subsequently stored at -80 degrees C until further investigation. | CLSI | Not clear which samples are CSF or blood. Total numbers of bacteria isolated not reported.  Missing data. Sampling strategy not well defined. Bloods collection and processing infomation not given, Number of excluded cases not included. Not differentiate between CA/HA. Duplicates not reported. MDR not defined. | D | Observational study.  Sampling strategy not clear. Small sample size. Missing data. Significant risk of bias. | E | Overt ID and/or AST errors detected/strongly suspected |
| --- | --- | --- | --- | --- | --- | --- | --- | --- | --- | --- | --- | --- | --- | --- | --- | --- | --- | --- | --- | --- |

| **Qu, L** | 2016 | Pathogen and antimicrobial resistance profiles of culture-proven neonatal sepsis in Southwest China, 1990-  2014. | Case series | Retrospective | China | Urban tertiary | 0 | 966 | 87543 | N/A | Blood | *Escherichia coli Klebsiella* spp.  *Acinetobacte r* spp.  *Staphylococc us aureus* | 321 | Blood samples of approximately 0.5−1 mL were obtained from a peripheral vein after skin disinfection with  povidone- iodine.  Incubation was continued until a positive result was observed or up to a maximum of 5–7 days, at the end of which all the negative samples were subcultured once on blood agar before discarding.  The isolated organisms and their patterns of susceptibility to various antibiotics were recorded, based on routine laboratory testing. No methodology or reference included for organism ID. | N/A | Number of excluded cases not included.  Duplicates not reported. Internal inconsistency of numbers or isolates. Small number, single site. No information on Target Org ID or AST methodology. | D | Observational study. Small sample size. Significant risk of bias. | E | Overt ID and/or AST errors detected/strongly suspected |
| --- | --- | --- | --- | --- | --- | --- | --- | --- | --- | --- | --- | --- | --- | --- | --- | --- | --- | --- | --- | --- |

| **Arushothy, R** | 2019 | Pneumococcal serotype distribution and antibiotic susceptibility in Malaysia: A four-year study (2014-2017) on  invasive paediatric isolates | Case series | Prospective | Malaysi a | Urban tertiary with samples received from hospital s around the country (no informa tion on these provide d). | 0-5 | 245 | 1847  (includes non invasive and adult isolates) | N/A | Blood CSF | *Streptococcu s pneumoniae* | 245 | No information on specimen processing.  Target ID via Gram staining, optochin sensitivity, and bile solubility tests. | CLSI | Not all hospitals in Malaysia participated in the surveillance. No further information about which hospitals included in study and their geographic coverage. | C | Observational study but over a 3 years wth multiple sites and prospective data collection.  Included clear case definitions and AST methodology. Significant risk of bias. | D | Accreditation details not provided. EQA participation not confirmed. Organism ID and AST methodology partially described. No ID or AST errors detected |
| --- | --- | --- | --- | --- | --- | --- | --- | --- | --- | --- | --- | --- | --- | --- | --- | --- | --- | --- | --- | --- |

| **Wang, S** | 2018 | Clinical characteristics of nosocomial bloodstream infections in neonates in two hospitals, China. | Case series | Retrospective | China | Urban tertiary | 0 | 530 | 43146 | HA | Blood | *Streptococcu s pneumoniae Escherichia coli Klebsiella* spp.  *Staphylococc us aureus* | 242 | Vitek (bioMerieux, Hazelwood, MO).  Specimens cultured on Sabouraud dextrose agar and chocolate agar plates in the presence of antibiotics to identify potential fungal pathogens and were further analyzed by microscopy (germ tube formation in horse serum) and biochemical API20C AUX  and/or API 32C  (BioMe´rieux) assays. | CLSI | Sampling strategy not well defined. MDR not reported. | D | Observational study over a 10 years with  2 sites. Sampling strategy not well defined. Significant risk of bias. | E | Overt ID and/or AST errors detected/strongly suspected |
| --- | --- | --- | --- | --- | --- | --- | --- | --- | --- | --- | --- | --- | --- | --- | --- | --- | --- | --- | --- | --- |

| **Shi, W** | 2019 | Serotype distribution, antibiotic resistance pattern, and multilocus sequence types of invasive Streptococcus pneumoniae isolates in two tertiary pediatric hospitals in Beijing prior to PCV13  availability. | Case series | Not stated - presumed retrospective | China | Urban tertiary | 0-14 | 111 | N/A | N/A | Blood CSF  Pleural effusion Subdural effusion Bone marrow | *Streptococcu s pneumoniae* | 111 | N/A | CLSI | Sampling strategy not defined.  Number of excluded cases not included.  Denominator not included. Not differentiate between CA/HA. Specimen processing / target Iorg ID process for IPD not included. | D | Observational study.  Sampling strategy not clear. Small sample size. Significant risk of bias. | E | Overt ID and/or AST errors detected/strongly suspected |
| --- | --- | --- | --- | --- | --- | --- | --- | --- | --- | --- | --- | --- | --- | --- | --- | --- | --- | --- | --- | --- |
| **Pan, T** | 2020 | Late-onset neonatal sepsis in Suzhou, China. | Case series | Retrospective | China | Urban tertiary | 0 | 190 | 213 | N/A althoug h narrativ e suggest HA | Blood CSF | *Staphylococc us aureus Escherichia coli Streptococcu s agalactiae Klebsiella* spp. | 133 | VITEK 2 drug sensitivity analyzer (BioMerieux, France). | N/A | MDR not defined. AMR not defined. | C | Observational study single site over 7 year period. Large number.  Significant risk of bias. | E | Overt ID and/or AST errors detected/strongly suspected |

| **Ma, X** | 2013 | Serotype Distribution and Antimicrobial Resistance of Streptococcus pneumoniae Isolates Causing Invasive Diseases from Shenzhen Children's Hospital | Case series | Not stated - presumed retrospective | China | Urban tertiary | 0-13 | 89 | N/A | N/A | Blood CSF  abscess aspiration Pleural effusion Joint fluid Abdominal fluid Bronchioalve olar lavage fluid | *Streptococcu s pneumoniae* | 89 | All isolates were stored at  -80 degrees C in a fat-free milk preservation medium until further analysis. All isolates were typed by a capsule- quelling test using type- specific antisera (Statens Serum Institute, Copenhagen, Denmark) against the serotypes present in the 23-valent pneumococcal polysaccharide vaccine (1, 2,  3, 4, 5, 6B, 7F,  8, 9N, 9V, 10A,  11A, 12F, 14,  15B, 17F, 18C,  19A, 19F, 20,  22F, 23F, and  33F) and serotype 6A. Typing was conducted by phase-contrast microscopy according to the published procedure. | CLSI | Sampling strategy not defined.  Number of excluded cases not included.  Denominator not included. Not differentiate between CA/HA. Duplicates not reported. | D | Observational study.  Sampling strategy not clear. Small sample size. Significant risk of bias. | D | Accreditation details not provided. EQA participation not confirmed. Organism ID and AST methodology partially described. No ID or AST errors detected |
| --- | --- | --- | --- | --- | --- | --- | --- | --- | --- | --- | --- | --- | --- | --- | --- | --- | --- | --- | --- | --- |

| **Peng, X** | 2021 | Prevalence and antimicrobial resistance patterns of bacteria isolated from cerebrospinal fluid among children with bacterial meningitis in China from 2016 to 2018: a  multicenter retrospective study | Case series | Retrospective | China | Urban tertiary and urban- non tertiary | 0-17 | 1142 | N/A | N/A | CSF | *Escherichia coli Klebsiella* spp.  *Acinetobacte r* spp.  *Staphylococc us aureus Streptococcu s pneumoniae Pseudomona s aeruginosa Streptococcu s agalactiae* | 462 | All participating hospitals strictly complied with the standard operating procedures for CSF collection and culture. According to the Clinical Laboratory Standards Institute (CLSI) guidelines, local experienced laboratory members of each hospital independently completed the isolation and identification of isolates. | CLSI | Sampling strategy not defined.  Number of excluded cases not included.  Denominator not included. Not differentiate between CA/HA. Duplicates not reported. | C | Observational study multiple sites over 2 year period. Large number.  Significant risk of bias. | E | Overt ID and/or AST errors detected/strongly suspected |
| --- | --- | --- | --- | --- | --- | --- | --- | --- | --- | --- | --- | --- | --- | --- | --- | --- | --- | --- | --- | --- |

| **Tang, X** | 2020 | Changing trends in the bacteriological profiles and antibiotic susceptibility in neonatal sepsis at a tertiary children's hospital of China | Case series | Retrospective | China | Urban tertiary | 0 | 1479 | N/A | CA  presum ed | Blood | *Escherichia coli Klebsiella* spp.  *Acinetobacte r* spp.  *Staphylococc us aureus Salmonella* spp. | 332 | Blood from peripheral vein under aseptic conditions before antibiotic interventions. Blood (2 mL) was inoculated into the brain- heart infusion broth at 37 ℃.  Sub-cultures  were produced on blood and MacConkey’s agar following 24 and 48 h of growth.  Bacterial growth was identified through colony counting, Gram staining, slide agglutination, and biochemical properties. If no growth was evident after 48 h, then the growth period was extended to 7 days before being confirmed as sterile. Mixed bacterial flora or diphtheroid growth represented contamination  . Coagulase- negative Staphylococci (CoNS) was considered pathogen only when isolated in paired cultures. | CLSI | Number of excluded cases not included.  Denominator not included.  Duplicates not reported. Single centre small size. Streptococcus a major pathogen 5% but not delineate the subspecies. | C | Observational study. Large sample size. Risk of bias. | C | Accreditation details not provided. EQA participation not confirmed. Organism ID and AST methodology completely described. No ID or AST errors detected |
| --- | --- | --- | --- | --- | --- | --- | --- | --- | --- | --- | --- | --- | --- | --- | --- | --- | --- | --- | --- | --- |

| **Li, X** | 2019 | Clinical features and antimicrobial susceptibility profiles of culture-proven neonatal sepsis in a tertiary children's hospital, 2013  to 2017. | Case series | Retrospective | China | Urban tertiary | 0 | 341 | 3454 | Most CA | Blood CSF | *Streptococcu s agalactiae Escherichia coli Klebsiella* spp.  *Acinetobacte r* spp.  *Staphylococc us aureus* | 91 | N/A | CLSI | Single centre. Specimen colleciton/ processing not described.  Strep.spp grouped together.  Alcaligenes xylosoxidans the major Gram neg isolated and contributing to 40% of EOS- how can this be interpreted as usually a hospital acquired infection? | D | Observational study. Large number excluded due to missing data.  Specimen collection/pro cessing not clear.  Significant risk of bias. | E | Overt ID and/or AST errors detected/strongly suspected |
| --- | --- | --- | --- | --- | --- | --- | --- | --- | --- | --- | --- | --- | --- | --- | --- | --- | --- | --- | --- | --- |

| **Wang, X** | 2018 | Molecular characteristics of community- associated Staphylococcus aureus isolates from pediatric patients with bloodstream infections between 2012  and 2017 in Shanghai, China. | Case series | Retrospective | China | Urban tertiary | 0-17 | 78 | N/A | CA | Blood | *Staphylococc us aureus* | 78 | Gram stain and catalase and coagulase activity with rabbit plasma. API-Staph test (bioMérieux, Lyon, France). MRSA isolates were initially identified using cefoxitin screening and the presence of the mecA gene was confirmed by PCR. These isolates were processed in Class II Biological Safety Cabinets. All strains were stored at -70 degrees C and grown overnight on sheep blood agar plates at 37 degrees C. | CLSI | Sampling strategy not defined.  Number of excluded cases not included.  Denominator not included.  Duplicates not reported. MDR not defined. | D | Observational study.  Sampling strategy not well defined. Small sample size.  Significant risk of bias. | D | Accreditation details not provided. EQA participation not confirmed. Organism ID and AST methodology partially described. No ID or AST errors detected |
| --- | --- | --- | --- | --- | --- | --- | --- | --- | --- | --- | --- | --- | --- | --- | --- | --- | --- | --- | --- | --- |

| **Qiu, Y** | 2020 | Microbiological profiles and antimicrobial resistance patterns of pediatric bloodstream pathogens in China, 2016-  2018 | Case series | Retrospective | China | Urban tertiary | 0-17 | 9235 | 10046 | N/A | Blood | *Escherichia coli Klebsiella* spp.  *Acinetobacte r* spp*.*  *Staphylococc us aureus Streptococcu s pneumoniae Salmonella* spp*.*  *Pseudomona s aeruginosa Streptococcu s agalactiae* | 3241 | Limited specific detail: Species identification and antimicrobial susceptibility testing were performed at local hospital laboratories by automated systems such as Vitek or Phoenix, according to CLSI  performance guideline. | CLSI | Staph aureus not tested to first line agents.  Sensitive isolates number includes all the intermediate sensitive isolates (no number of total intermediate).  Large number CoNS unable to determine % contaminant. Unable to assess HA v CA. Limited clinical data to correlate significance of bloodstream infection with patient primary diagnosis. | B | Large sample size across 13 tertiary units. Risk of bias remains due to obersvational study. | D | Accreditation details not provided. EQA participation not confirmed. Organism ID and AST methodology partially described. No ID or AST errors detected |
| --- | --- | --- | --- | --- | --- | --- | --- | --- | --- | --- | --- | --- | --- | --- | --- | --- | --- | --- | --- | --- |

| **Jiang, Y** | 2016 | The clinical characteristics of neonatal sepsis infection in Southwest China. | Case series | Retrospective | China | Urban tertiary | 0 | 133 | 2765 | N/A | Blood | *Escherichia coli Klebsiella* spp.  *Acinetobacte r* spp.  *Staphylococc us aureus Streptococcu s agalactiae* | 53 | On admission, 1-3 mL of venous blood was directly drawn into a BACT/ALERT  specimen collection tube (BioMérieux, Lyons, France) before patients received any antibiotic treatment.  Blood cultures were performed using the fully automated BACT/ALERT 3 D-120  microbial detection system BioMérieux). ID with BBL CrystalTM Identification Systems (BD Diagnostics, Sparks, USA) and APIⓇ strips (BioMérieux) prior to June 2010. VITEK 2  after June 2010. | CLSI | Single site, small sample size.  Unable to assess HA v CA. MDR not reported. Most CONS. Risk of contamination controlled for by requiring x2 +ve BC to make diagnosis of CONS and correlation with clinical data suggestive of sepsis. | D | Observational study. Risk of bias. Errors in reporting as only give % not number tested and Res/sensitive. | C | Accreditation details not provided. EQA participation not confirmed. Organism ID and AST methodology completely described. No ID or AST errors detected |
| --- | --- | --- | --- | --- | --- | --- | --- | --- | --- | --- | --- | --- | --- | --- | --- | --- | --- | --- | --- | --- |
