# Supplementary material for: Coverage gaps in empiric antibiotic regimens used to treat serious bacterial infections in neonates and children in Southeast Asia and the Pacific
Source: Lancet Reg Health Southeast Asia. 2023 Oct 31;22:100291. doi: 10.1016/j.lansea.2023.100291 (PMC10934317; doi:10.1016/j.lansea.2023.100291)
Supplement: Supplementary Table 3 [file mmc3.docx]

# Supplementary Table 3

Intrinsic resistance assumptions, including bug-drug combinations whereby a lack of *in vivo* activity (despite *in vitro*) activity is known.

| **Pathogen** | **Intrinsic Resistance /**  **Lack of in vivo activity Assumptions** |
| --- | --- |
| *Enterococcus* spp. | Cephalosporins  Macrolides  Flucloxacillin / methicillin  Gentamicin / aminoglycosides as monotherapy |
| *Klebsiella* spp. | Ampicillin  Flucloxacillin / methicillin  Vancomycin / glycopeptides  ***Klebsiella aerogenes:***  Amoxicillin-clavulanic acid  Ampicillin-sulbactam  Cefazolin, cefalexin, cefoxitin |
| *Acinetobacter* spp. | Aminopenicillin  Ampicillin-clavulanic acid  Ceftriaxone, cefotaxime* as per EUCAST^28^  Ertapenem  Trimethoprim  Tetracycline, doxycycline  Vancomycin / glycopeptides  Flucloxacillin / methicillin |
| *Pseudomonas aeruginosa* | Aminopenicillin  Amoxicillin-clavulanic acid  Ampicillin-sulbactam  Ceftriaxone/cefotaxime  Chloramphenicol  Trimethoprim  Tetracyclines  Vancomycin / glycopeptides  Flucloxacillin / methicillin |
| *Streptococcus pneumoniae* | Gentamicin |
| *Escherichia coli* | Flucloxacillin / methicillin  Vancomycin / glycopeptide |
| *Salmonella* spp. | Gentamicin / aminoglycosides  Vancomycin / glycopeptides  Flucloxacillin / methicillin |
| Enterobacterales not otherwise specified above | Vancomycin / glycopeptides  Flucloxacillin / methicillin |
