# Supplementary material for: Coverage gaps in empiric antibiotic regimens used to treat serious bacterial infections in neonates and children in Southeast Asia and the Pacific
Source: Lancet Reg Health Southeast Asia. 2023 Oct 31;22:100291. doi: 10.1016/j.lansea.2023.100291 (PMC10934317; doi:10.1016/j.lansea.2023.100291)
Supplement: Supplementary Table 4 [file mmc4.docx]

# Supplementary Table 4 - Model specifications

| **Pathogen distribution** | For the distribution of pathogens, we adopted the commonly used Dirichlet / Multinomial conjugate model. The distributions of causative pathogens associated with each syndrome were modelled independently.  $y\sim\text{Multinomial(}\theta)$ $\theta\sim\text{Dirichlet}\left( a \right)$ $a = 1\forall k$  where $y\in\mathbb{N}^{K}$ denotes the total number of isolates tested for each organism $k=1...K$ and with and $\theta\in K \text{simplex}$ for a given syndrome.  For each of the syndromes, each paper reported the total number of isolates by organism, which when pooled implied a crude estimate of the distribution of pathogens. However, a limitation of this model is that variability is inherent in the total numbers of isolates by organism across each publication source, which is not accounted for in the above. |
| --- | --- |
| **Susceptibility model** | Hierarchical logistic regression was used to estimate the organism susceptibility to antibiotics. Each organism was modelled independently.  $y_{i}\sim\text{Binomial}\left( n_{i},\pi_{i} \right)$ $\text{logit}\left( \pi_{i} \right)=\beta_{\text{class}\left[ i \right]}+z_{\text{ab}\left[ i \right]}\sigma_{\text{ab}}+z_{\text{pub}\left[ i \right]}\sigma_{\text{pub}}$ $\beta_{\text{ab}\left[ i \right]}\sim\text{Normal(}\text{logit}^{-1}(0.6), 3)$ $z_{\text{ab}}\sim\text{Normal}\left( 0, 1 \right)$ $z_{\text{pub}}\sim\text{Normal}\left( 0, 1 \right)$ $\sigma_{\text{ab}}\sim\text{Exponential}\left( 2 \right)$ $\sigma_{\text{pub}}\sim\text{Exponential(2)}$  where $y_{i}$ denotes the number of isolates susceptible out of $n_{i}$ for the $i^{th}$ observation that amounted to a stratification of the data by antibiotic class, antibiotic, and publication for the organism under consideration. The terms within the linear predictor correspond to antibiotic class intercepts $\beta_{\text{class}[i]}$ and variance components for antibiotic and publication ($z_{\text{ab}\left[ i \right]}\sigma_{\text{ab}}$ and $z_{\text{pub}\left[ i \right]}\sigma_{\text{pub}}$ respectively) which we expand upon below.  For each of the organisms considered, there are a series of antibiotic classes that would commonly be used in treatment. For example, in the case of *E. coli*, the treatment options would normally span 3GC, aminopenicillin, carbapenems and gentamicin. Within each of these classes multiple antibiotics might be represented in the data, such as ceftriaxone or cefotaxime for 3GC, and within a given class, we would (generally) expect each of the antibiotics to be exchangeable, each having a comparable level of action against a target organism. However, the data also originated from a diverse set of publications, which we would anticipate would also show heterogeneity; paper 1 might report that for cefotaxime 13 out of 25 isolates were susceptible, paper 2 might report that 6 of 27 were susceptible and so on. Rather than simply aggregate all the data together and ignore this heterogeneity, we model it explicitly via random effects for antibiotic and the publication source. We therefore have components in the linear predictor for the class of antibiotic, $\beta_{\text{class}[i]}$ a variance component for antibiotic type, $\sigma_{\text{ab}}$ and a variance component for publication/source of information, $\sigma_{\text{pub}}$. The goal is to partially-pool across all the sources of variability and to allow us to obtain estimates for the susceptibility of an organism to each specific antibiotic in a manner that leverages other data within the relevant classes. In aggregate, the priors are consistent with a median susceptibility of 0.6 on the probability scale with concentration on the boundaries. That is, the *a-priori* baseline risk is assumed to adopt any value across the probability scale. |
