# Supplementary figures and images for: Coverage gaps in empiric antibiotic regimens used to treat serious bacterial infections in neonates and children in Southeast Asia and the Pacific
Source: Lancet Reg Health Southeast Asia. 2023 Oct 31;22:100291. doi: 10.1016/j.lansea.2023.100291 (PMC10934317; doi:10.1016/j.lansea.2023.100291)

Supplementary Figure 1. PRISMA flow chart

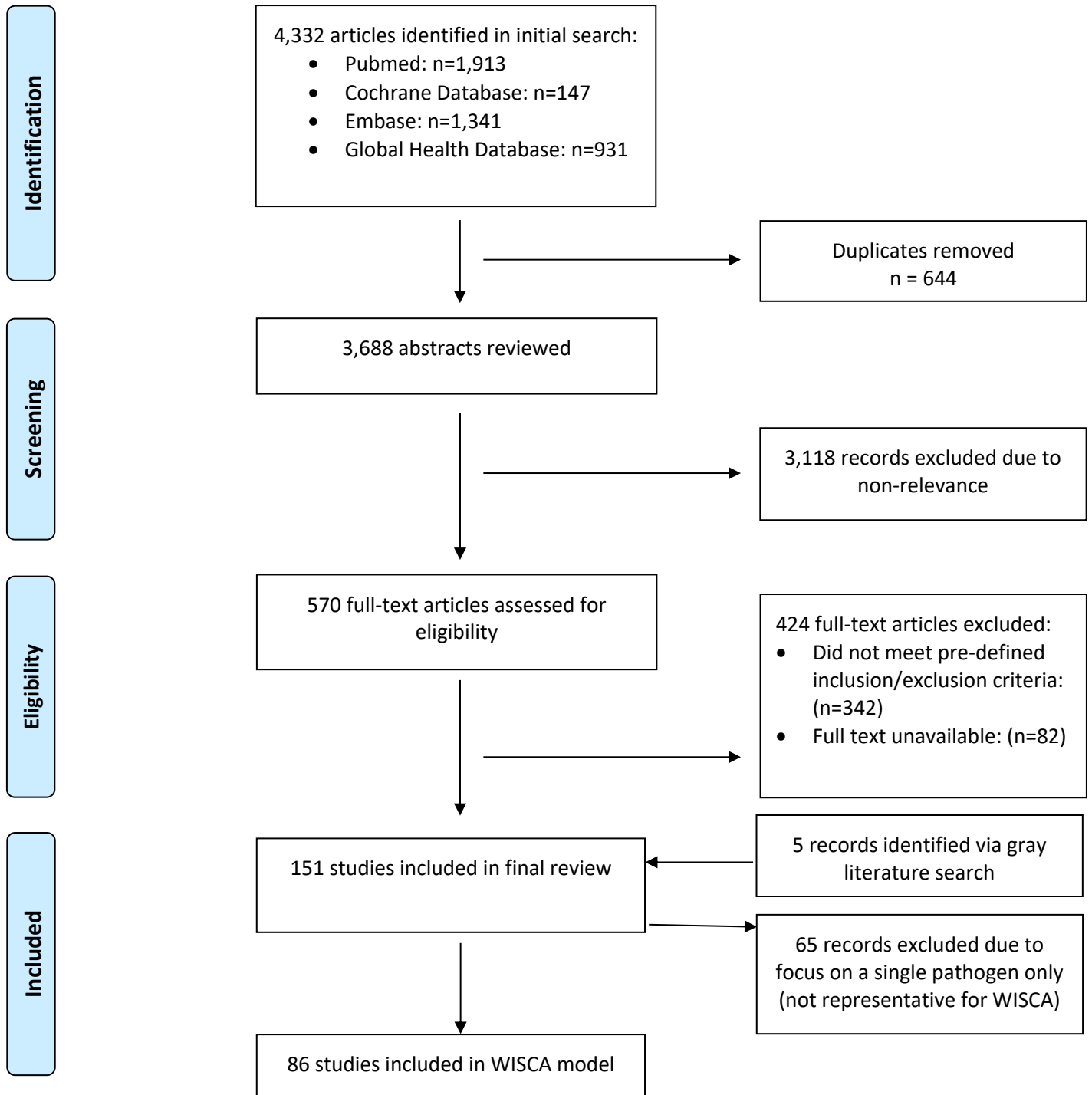

Supplement: Supplementary Figure 1 [file mmc5.pdf]
